# Functional integration of a semi-synthetic azido-queuosine derivative into translation and a tRNA modification circuit

**DOI:** 10.1093/nar/gkac822

**Published:** 2022-09-28

**Authors:** Larissa Bessler, Navpreet Kaur, Lea-Marie Vogt, Laurin Flemmich, Carmen Siebenaller, Marie-Luise Winz, Francesca Tuorto, Ronald Micura, Ann E Ehrenhofer-Murray, Mark Helm

**Affiliations:** Institute of Pharmaceutical and Biomedical Sciences, Johannes Gutenberg-University Mainz, 55128 Mainz, Germany; Institute of Biology, Humboldt-Universität zu Berlin, 10117 Berlin, Germany; Institute of Pharmaceutical and Biomedical Sciences, Johannes Gutenberg-University Mainz, 55128 Mainz, Germany; Department of Organic Chemistry, University of Innsbruck, 6020 Innsbruck, Austria; Department of Chemistry – Biochemistry, Johannes Gutenberg-University Mainz, 55128 Mainz, Germany; Institute of Pharmaceutical and Biomedical Sciences, Johannes Gutenberg-University Mainz, 55128 Mainz, Germany; Division of Biochemistry, Mannheim Institute for Innate Immunoscience (MI3), Medical Faculty Mannheim, Heidelberg University, Mannheim, Germany; Department of Organic Chemistry, University of Innsbruck, 6020 Innsbruck, Austria; Institute of Biology, Humboldt-Universität zu Berlin, 10117 Berlin, Germany; Institute of Pharmaceutical and Biomedical Sciences, Johannes Gutenberg-University Mainz, 55128 Mainz, Germany

## Abstract

Substitution of the queuine nucleobase precursor preQ_1_ by an azide-containing derivative (azido-propyl-preQ_1_) led to incorporation of this clickable chemical entity into tRNA *via* transglycosylation *in vitro* as well as *in vivo* in *Escherichia coli*, *Schizosaccharomyces pombe* and human cells. The resulting semi-synthetic RNA modification, here termed Q-L1, was present in tRNAs on actively translating ribosomes, indicating functional integration into aminoacylation and recruitment to the ribosome. The azide moiety of Q-L1 facilitates analytics *via* click conjugation of a fluorescent dye, or of biotin for affinity purification. Combining the latter with RNAseq showed that TGT maintained its native tRNA substrate specificity in S*. pombe* cells. The semi-synthetic tRNA modification Q-L1 was also functional in tRNA maturation, in effectively replacing the natural queuosine in its stimulation of further modification of tRNA^Asp^ with 5-methylcytosine at position 38 by the tRNA methyltransferase Dnmt2 in *S. pombe*. This is the first demonstrated *in vivo* integration of a synthetic moiety into an RNA modification circuit, where one RNA modification stimulates another. In summary, the scarcity of queuosinylation sites in cellular RNA, makes our synthetic q/Q system a ‘minimally invasive’ system for placement of a non-natural, clickable nucleobase within the total cellular RNA.

## INTRODUCTION

Post-transcriptional modification of tRNAs is a ubiquitous yet idiosyncratic feature with versatile chemical structures contributing to stability and folding, as well as fidelity of decoding and translational control (1–3). The largest variety of chemical structures in RNA is found in the anticodon loop which directly interacts with the mRNA during decoding in the translating ribosome. The chemical variety of the more than 170 modifications known to date is dominated by tRNA anticodon modifications occurring at positions 34 and 37, ranging from simple methylations to highly complex structures of which queuosine (Q) is a particular case (4,5). In both, prokaryotes and eukaryotes, this hypermodified 7-deazaguanosine is exclusively found in the anticodon wobble position 34 of tRNAs containing a G_34_U_35_N_36_ motif and therefore specific for a selected group of four tRNAs, namely tRNA^Asn^, tRNA^Asp^, tRNA^His^ and tRNA^Tyr^ (6,7). In an intricate multi-step process involving various enzymes and co-factors, *Escherichia coli* and other prokaryotes are capable of first synthesising the modified precursor base 7-aminomethyl-7-deazaguanine (preQ_1_) *de novo*. GTP is converted to preQ_1_*via* five enzymatic steps successively catalysed by the GTP cyclohydrolase I (GCH I), QueD, QueE, QueC and QueF (8–11). As a rare type of post transcriptional modification, the noncanonical nucleobase structure is then introduced into tRNA in an exchange reaction. During this transglycosylation step, the bacterial tRNA guanine transglycosylase (bTGT) replaces a guanosine in the anticodon wobble position of cognate tRNAs with the precursor base preQ_1_ (12,13) which is then further enzymatically modified by QueA and QueG to yield the final queuosine structure (14,15). In contrast to prokaryotes, eukaryotes salvage the nucleoside queuosine and the corresponding nucleobase queuine (q) from environmental sources including the gut microbiota (reviewed in (5)). Queuosine is hydrolyzed by a queuosine nucleoside glycosylase to release q (16). The incorporation of the salvaged q into tRNA is catalysed by eukaryotic TGT (eTGT), which is a heterodimeric enzyme (17–19) composed of a catalytic queuine tRNA-ribosyltransferase subunit 1 (QTRT1) and a noncatalytic queuine tRNA-ribosyltransferase subunit 2 (QTRT2) (20).

Despite its suggestive positioning at position 34 of the tRNA anticodon, molecular details of the physiological relevance of Q remain scarce. It is generally accepted that Q impacts the decoding process on the translating ribosome, with cumulative evidence pointing to pivotal interactions at the A-site. The specific occurrence of Q in GUN anticodons is consistent with a general concept by Grosjean and Westhof (21), wherein modifications at position 34 compensate for the lower stability of codon-anticodon interactions including 2 or more base pairs with less than three hydrogen bonds. This concept receives support from computational modelling, which characterised a stabilising effect of Q on the overall tRNA-mRNA complex involving additional hydrogen bonds (22). *In vivo* and *in cellulo* studies did not reveal any strong phenotypes under Q deficiency or in strains lacking TGT. However, the presence of Q improved viability under stress conditions and affected translation accuracy in *E. coli* (23,24). *In vivo* studies in eukaryotes likewise reported an impact on the decoding process, enabling decoding of synonymous codons by wobble base pairing (22,25,26), and affecting translation speed and accuracy (27,28). Queuosine's multifaceted involvement in the cellular machinery was reported to be associated with cancer (29–32), neuronal disorders (33–35) as well as bacterial and parasitic infection (36,37). Consequently, the perception of therapeutic potential associated with its biogenesis has consistently increased, in keeping with a general trend in epitranscriptomics

So far, the only demonstrated molecular interaction affected by Q outside the ribosome is a so-called modification circuit with 5-methylcytidine (m^5^C) in position 38 of *Schizosaccharomyces pombe* tRNA^Asp^, stimulating its formation by the Dnmt2 homologue Pmt1 (38,39). Structural analysis suggested that the presence of Q34 leads to optimal positioning of the interacting substrates in the active site of Dnmt2, enhancing the catalytic efficiency of the methyltransferase (40).

Arguably, approaches to a deeper understanding of the molecular action of Q in living cells would need to involve manipulations of details of the structure of Q, e.g. *via* an incorporation of q-derivatives through transglycosylation. Apart from their natural substrates, both bTGT and eTGT have been shown to tolerate a certain variety of synthetic analogues harbouring large functional groups *in vitro* (41,42). Leveraging the short hairpin recognition motif of the bTGT installed on different RNA transcripts, Devaraj and co-workers developed a method called RNA-TAG (transglycosylation at guanosine), allowing to site-specifically incorporate analogues *in vitro*, which contained large fluorophores or affinity labels for pull-down experiments. This method was also applied to visualize mRNA transcripts containing the recognition motif in a fixed cell environment in a direct one-step-reaction (42) and extended to the development of a light-activated mRNA translation system (43). Furthermore, RNA-TAG was used on modified mRNAs in a two-step-approach, incorporating a preQ_1_-derivative bearing a bioorthogonal tetrazine moiety in the first step, and thus enabling further derivatization by IEDDA click chemistry in a second step (44). However, labelling with click-competent compounds *in vivo* or *in cellulo* has not yet been achieved in the queuosine field. Indeed, there is strongly suggestive, albeit indirect evidence of successful *in vivo* incorporation of a non-natural q-analogue as published by Kelly and co-workers in the context of an animal model of multiple sclerosis (34). In addition to concerns about cell permeability of a q-derivative, important aspects to determine for *in vivo* labelling studies would include the physiological impact of an artificial chemical structure in a functioning tRNA, which would primarily be expected on the level of translation.

In this study, we metabolically label tRNA with a preQ_1_ derivative functionalized with an azide group, allowing for further derivatization by click reaction and thus facilitating the proof of successful incorporation as well as the isolation of accordingly tagged RNAs. The latter was combined with RNAseq, in order to re-investigate the RNA substrates of the TGT, which turned out to be specific for the previously reported tRNAs Asn, Asp, His and Tyr. While in previous studies the transglycosylation step was performed in a fixed cell environment, we herein focus on the incorporation of the analogues by the natively expressed TGT *in vivo* and the physiological consequences in the natural environment. Polysome preparations revealed an enrichment of Q-containing tRNAs in the polysomal fraction, indicating a targeted selection for modified tRNA to be integrated in the translational process. Moreover, our data demonstrate that the semi-synthetic tRNA modification replaces Q34 and is functionally integrated into the translational process, as well as in the modification circuit with m^5^C38 in tRNA^Asp^ in *S. pombe*.

## MATERIALS AND METHODS

*S. pombe* strains used in this study are given in Supplementary Table S1, Plasmids used in this study are given in Supplementary Table S2, oligonucleotides used in this study are given in Supplementary Table S3. The names and versions of all software used are provided in Supplementary Table S4.

### Synthesis of preQ_1_-L1, preQ_1_-L2 and preQ_1_-L3

The preQ_1_-ligands were synthesized as previously described (45).

### Recombinant expression and purification of bTGT

The pASK-IBA13plus vector expressing the *Zymomonas mobilis* TGT (bTGT) with a N-terminal Strep-tag II was kindly provided by Prof. Dr Klaus Reuter (Philipps-University, Marburg). Expression and purification were carried out as previously described with minor changes (46). Briefly, the TGT was expressed in *E. coli* BL21-CodonPlus (DE3)-RIPL cells, grown in 2× YT medium and protein production was induced using anhydrotetracycline to a final concentration of 0.2 mg/l. After growing the cells for 14 h at 15°C, cells were harvested and the cell pellets were stored at –80°C until further processing. To purify the bacterial TGT, cells were thawed in lysis buffer (100 mM Tris pH 7.8, 150 mM NaCl, 1 mM EDTA pH 8.0, 2 mM PMSF, 1 μg/ml leupeptin, 1 μg/ml aprotinin, 1 μg/ml pepstatin and 25 U of DNase I and RNase I, respectively). After sonication (60% amplitude, 6 min, 0.5 s on, 2 s off; Sonifier 250 D, Branson), soluble proteins were isolated by centrifugation at 20 000 g for 1 h, 4°C. Affinity chromatography was then used to purify the Strep II-tagged TGT. For this purpose, the lysate was incubated with Strep-Tactin^®^ Superflow Plus resin (Qiagen) for 3 h at 4°C, 15 rpm. After washing with washing buffer (100 mM Tris pH 7.8, 150 mM NaCl, 1 mM EDTA pH 8.0), the protein complex was eluted in 100 mM Tris pH 7.8, 150 mM NaCl, 1 mM EDTA pH 8.0 and 2.5 mM desthiobiotin. Further purification was achieved by Superdex S200 (GE Healthcare) size exclusion chromatography (10 mM Tris pH 7.8, 150 mM NaCl, 1 mM EDTA pH 8.0). The purified bTGT was stored at –80°C in 10 mM Tris pH 7.8, 150 mM NaCl, 1 mM EDTA pH 8.0 with 50% glycerol.

### *E. coli* strains and growth conditions

The *E. coli* Keio parent strain (BW25113) and the knockout strains for QueD, QueC, QueE, QueF and TGT were obtained from the *E. coli* Keio knockout collection (GE Healthcare (Dharmacon™), England) and grown in standard M9 medium (6.8 g/l Na_2_HPO_4_, 3 g/l KH_2_PO_4_, 0.5 g NaCl, 1 g/l NH_4_Cl, 2 mM MgSO_4_, 0.1 mM CaCl_2_, 0.4% glucose) at 37°C and 190 rpm. Growth medium of knockout strains was additionally supplemented with kanamycin (25 μg/ml). Synthetic preQ_1_-derivatives were added to final concentrations of 0.1, 1, 5 or 10 μM to the culture, respectively.

### Isolation of total tRNA from *E. coli*

To isolate total tRNA, *E. coli* cells were grown to an OD_600_ of 1 in 50 ml cultures and harvested by centrifugation (10 min, 10 000 g, 4°C). The RNA was extracted by using the RNA isolation reagent TRI Reagent^©^ (Sigma-Aldrich, Germany) following the manufacturer's instructions and dissolved in MQ-water.

### Polysome preparations from *E. coli*

For polysome preparations the *E. coli* cells were grown in M9 medium in 150 ml culture volume until they reached an OD of 0.6, chloramphenicol was added to final concentration of 100 μg/ml and after further incubation of 3 min the cells were harvested by centrifugation (10 min, 10 000 g, 4°C). For cell lysis, cell pellets were resuspended in buffer (100 mM NH_4_Cl, 10 mM MgCl_2_, 20 mM Tris, pH 7.5), lysozyme was added and freeze-thaw cycles in liquid nitrogen were performed. Subsequent to this 10% deoxycholate was added to complete lysis, remaining cell wall debris were separated by centrifugation (12 000 g, 10 min, 4°C). Sucrose gradients from 5 to 40% were generated using a Biocomp gradient station model 108 (settings: time 1.24 min, angle 81.5°, speed 21 rpm) and lysate was loaded on top of the gradient. After ultracentrifugation (Beckman Ultracentrifuge Optima LE-80K, SW40 Ti rotor from Beckman Coulter) at 150 000 g and 4°C for 2.5 h, gradients were fractionated by measuring the absorbance at 280 nm (Biocomp Gradient Station model 108 in combination with Gilson Fraction Collector FC203B). Total RNA was isolated from the respective fractions using TRI reagent^©^ (Sigma-Aldrich).

### Purification of total tRNA from collected fractions by gel elution

Total RNA extracted from polysomal fraction was separated on a 10% denaturing PAGE gel, stained with GelRed (Biotium) and the bands were visualized on Typhoon 9400 at an excitation wavelength of 532 nm. According to the resulting image, bands of interest were excised from the gel and mashed with a scalpel. The mashed gel pieces were frozen for 1 h and 300 μl of 0.5 M ammonium acetate were added. Subsequently, the samples were shaken at 25°C and 750 rpm overnight. The gel suspension was filtered through NanoSep® centrifugal filters and the filtrate was precipitated with three volumes of 100% ethanol.

### *S. pombe* strains, plasmids and growth conditions

The *S. pombe* strains and plasmids used in this study are shown in Supplementary Table S1. Cells were cultured in YES (5 g/l yeast extract, 30 g/l glucose, 250 mg/l adenine, 250 mg/l histidine, 250 mg/l leucine, 250 mg/l uracil, 250 mg/l lysine) which did not contain queuosine or queuine. Synthetic queuine (kindly provided by Hans-Dieter Gerber and Gerhard Klebe (Universität Marburg) (47)) and preQ_1_ derivatives were added to 0.1 μM to the culture.

### Isolation of total RNA and small RNAs from *S. pombe*

To isolate total RNA, *S. pombe* cells were grown to an optical density at 600 nm (OD_600_) of 1 in 50 ml cultures. 50 OD of cells were harvested and 1 ml of phenol, glass beads were added. After vigorous shaking for 5 min, samples were centrifuged at 20 000 g for 5 min to clear the cell debris. Equal volume of phenol/chloroform/isoamylalcohol was added to the aqueous phase and centrifuged at 20 000 g for 5 min. After mixing the upper phase with an equal volume of chloroform followed by centrifugation at 20 000 g for 5 min, the RNA was precipitated at –80°C for 1 h using 0.7 volume of isopropyl alcohol. Following precipitation, total RNA was washed with 70% ethanol and eluted in DEPC-treated water.

Isolation of small RNAs was performed using the PureLink™ miRNA Isolation Kit (Invitrogen) according to the manufacturer's instructions. Yeast cells were grown to an OD_600_ of 1 in 5 ml cultures. After harvesting 1 OD of cells, RNAs were isolated using 1 ml TriFast reagent (Peqlab), 0.2 ml chloroform and glass beads. After vigorous shaking for 2 min, samples were centrifuged at 16 000 g, 4°C for 15 min. After adding 215 μl ethanol to the aqueous phase, the samples were transferred to a spin cartridge followed by centrifugation at 12 000 g for 1 min. 700 μl ethanol was added to the flow-through and the sample was transferred to a new spin cartridge. Following centrifugation at 12 000 g for 1 min, the cartridge was washed and small RNAs were dissolved in DEPC-treated water. Northern blot–acryloyl aminophenylboronic acid (APB) gels were performed as previously described (28).

### Removal of ribosomal RNA

Depletion of ribosomal RNA was performed as previously described (48). Oligonucleotides specific for 5.8S and 5S rRNA were ordered with a 5’-biotin tag from Metabion (see Supplementary Table S3). The oligonucleotides were diluted to 100 μM each in nuclease-free water and equal volumes of the 100 μM stock were combined to generate the rRNA depletion mix.

For hybridization, 8 μg of small RNAs were incubated with 9.92 μl of the 100 μM rRNA depletion mix in reaction buffer (10 μl of formamide, 2.5 μl of 20× SSC (3 M NaCl, 0.3 M sodium citrate, pH 7.0) and 5 μl of 0.005 M EDTA, pHnbsp;8). Reactions were carried out in a total volume of 50 μl with the following thermocycling: 80°C for 5 min, ramp down to 25°C at intervals of 5°C per minute. Following hybridization, 2 μl of RNase-OUT (Invitrogen) and 50 μl of 1× SCC containing 20% formamide were added. Removal of rRNA/oligonucleotide hybrids was performed using Dynabeads^TM^ MyOne^TM^ Streptavidin C1 (ThermoFisher) according to the manufacturer's instructions. 500 μl streptavidin coated magnetic beads were washed as instructed for immunoprecipitation of RNA and added to the hybridization reaction. After incubation for 15 min at room temperature with mild agitation and bead separation on a magnetic rack, the supernatant was once more incubated with 500 μl of washed beads for 15 min at room temperature under mild agitation followed by bead separation. Subsequently, the supernatant containing the 5S/5.8S rRNA-depleted RNA was precipitated with 1/10 volume of ammonium acetate and three volumes of 100% ethanol.

### RNA substrates for *in vitro* modification

The *S. pombe* tRNA^Asp^ substrate was prepared as previously described (49). Briefly, the pJET1 vector carrying the tRNA^Asp^ sequence was linearized with NcoI, and 2.5 μg of the linear vector was used for *in vitro* transcription using the TranscriptAid T7 High Yield Transcription Kit (Thermo Fisher Scientific) according to the manufacturer's instructions. Following an 8 h incubation at 37°C with nucleotides and the T7 RNA polymerase and subsequent DNase I treatment, the respective tRNA was purified from the reaction using phenol/chloroform extraction followed by gel filtration with Sephadex G50 (GE Healthcare).

### Recombinant expression and purification of hTGT

The pCDF-Duet1 vector co-expressing the human TGT (hTGT) heterodimer QTRT1 and QTRT2 with a cleavable N-terminal 6xHis tag to QTRT1 was kindly provided by Prof. Dr. Ralf Ficner (GZMB, Göttingen). Expression and purification were carried out as previously described with minor changes (20). Briefly, the heterodimer QTRT1/QTRT2 was co-expressed in *E. coli* (DE3) Rosetta cells, and protein production was induced using autoinduction. After growing the cells for 50 h at 18°C, cells were harvested and the cell pellets were stored at –80°C until further processing. To purify the human TGT, cells were thawed in lysis buffer (50 mM HEPES pH 7.5, 100 mM NaCl, 10 mM imidazole, 2 mM PMSF, 1 μg/ml leupeptin, 1 μg/ml aprotinin, 1 μg/ml pepstatin and 25 U of DNase I and RNase I, respectively). After sonification (60% amplitude, 6 min, 0.5 s on, 2 s off; Sonifier 250 D, Branson), soluble proteins were isolated by centrifugation at 20 000 g for 1 h. Affinity chromatography was then used to purify the 6xHis tagged QTRT1/QTRT2 complex. For this purpose, the lysate was incubated with Talon^®^ Superflow™ resin (Cytiva) for 3 h at 4°C, 15 rpm. After washing with washing buffer (50 mM HEPES pH 7.5, 100 mM NaCl, 10 mM imidazole and 1 M LiCl), the protein complex was eluted in 50 mM HEPES pH 7.5, 100 mM NaCl and 500 mM imidazole. Further purification was achieved by Superdex S200 (GE Healthcare) size exclusion chromatography (20 mM HEPES pH 7.5, 100 mM NaCl). The purified hTGT was stored at –80°C in 20 mM HEPES pH 7.5, 100 mM NaCl with 50% glycerol.

### *In vitro* labelling of tRNA with preQ_1_ derivatives

For *in vitro* labelling of tRNA with the preQ_1_ derivatives, 10 μM of *in vitro* transcribed tRNAs or alternatively 10 μg of total RNA from *S. pombe* was incubated with 200 nM hTGT (QTRT1:QTRT2) and 5 μM queuine in reaction buffer (50 mM Tris–HCl pH 7.5, 20 mM NaCl, 5 mM MgCl_2_ and 2 mM dithiothreitol) for 5 h at 37°C. The RNA was purified using phenol/chloroform extraction and precipitated with 1/10 volume of ammonium acetate and three volumes of 100% ethanol.

### HeLa cells growth conditions and *in vivo* labelling with preQ_1_-L1

HeLa cell lines were obtained from ATCC and authenticated by multiplex human cell line authentication test (Multiplexon). Cells were grown in Dulbecco's modified Eagle's medium (DMEM) (Thermo Fisher Scientific). The cultures were supplemented with 10% heat-inactivated FBS, 2 mM l-glutamine and a commercial cocktail of antibiotics (Thermo Fisher Scientific). For minus-Q conditions, ultraculture serum-free medium (Lonza) was supplemented with 2 mM l-glutamine and 100 units/ml Penicillin/Streptomycin. PreQ_1_-L1 derivative was added at a concentration of 0.1 μM for 72 h to the culture.

### HeLa cell polysome profiling

10^7^ cells were treated with 100 μg/ml cycloheximide for 5 min at RT to stabilize existing polysomes before washing with ice-cold PBS and harvesting by scraping in 400 μl polysome lysis buffer (20 mM Tris–HCl, pH 7.4, 5 mM MgCl_2_, 150 mM NaCl, 1 mM DTT, 1% Triton X-100, 100 μg/ml cycloheximide, 1 × Complete Protease Inhibitors (Roche)). Lysates were rotated end-over-end for 10 min at 4°C and cleared by at 10 000 rpm for 10 min at 4°C. 40 μl of supernatant lysate was saved as input before loading the lysates to linear 17.5 to 50% sucrose gradients in 20 mM Tris–HCl (pH 7.4), 5 mM MgCl_2_, 150 mM NaCl. Centrifugation was carried out at 35 000 rpm for 2.5 h at 4°C in a Beckmann SW60 rotor. Gradients were eluted with an ISCO UA-6 gradient fractionator, and polysome profiles were recorded by continuously monitoring the absorbance at 254 nm using PeakTrak software. During gradient elution, fractions of ∼300 μl were collected every 14 s. For RNA isolation, 300 μl urea buffer (10 mM Tris, pH 7.5, 350 mM NaCl, 10 mM EDTA, 1% SDS, and 7 M urea) and 300 μl phenol:chloroform:isoamylalcohol (25:24:1) were added to each fraction. After phase separation, RNA was isolated from the aqueous phase and precipitated using isopropanol and GlycoBlue (Thermo Fisher Scientific).

### CuAAC click reaction

Chemical clicking was performed as previously described (50). Briefly, up to 10 μg of RNA was incubated in reaction buffer containing 50% (v/v) DMSO, 5 mM Tris ((1-hydroxy-propyl-1*H*-1,2,3-triazol-4-yl)methyl) amine (THPTA), 5 mM sodium ascorbate, 0.5 mM CuSO_4_ and 50 μM ligand alkyne under light-protection for 2 h at 25°C. The ligand alkynes used were AlexaFluor 594 alkyne (Thermo Fisher Scientific) or biotin alkyne (PEG4 carboxamide-Propargyl biotin; Thermo Fisher Scientific). RNA was precipitated with 1/10 volume of ammonium acetate and three volumes of 100% ethanol.

### Detection of queuine and preQ_1_ modification of RNAs

Labelled RNA that had been CuAAC-clicked with AlexaFluor 594 alkyne was analyzed by denaturing PAGE. Up to 10 μg of labelled RNA was separated in 10% polyacrylamide gels (acrylamide/ bisacrylamide (19:1), urea 8 M in 1× TBE buffer). Detection was carried out on the Typhoon 9500 (GE Healthcare) using 532 nm for excitation. As a loading control, gels were stained with Sybr Gold nucleic acid gel stain (Thermo Fisher Scientific) or GelRed (Biotium) for 10 min followed by detection using 495 or 532 nm, respectively, for excitation.

To detect the queuine modification, 300 ng of total RNA or small RNAs from *S. pombe* WT and *qtr2Δ* strains were separated in a 10% polyacrylamide gel (acrylamide/ bisacrylamide (19:1), urea 8 M) supplemented with 5 mg/ml 3-(acrylamido)-phenylboronic acid as described previously (51). The separation was performed at room temperature in 1× TBE. The electrophoresed gels were transferred to a Biodyne B Nylon membrane (0.45 μM). Selected RNAs were detected using a 5′-biotin-labeled probe at a final concentration of 0.3 μM and the Chemiluminescence Nucleic Acid Detection Module Kit (Thermo Fisher Scientific) according to the manufacturer's instructions. The first blocking step was carried out using the DIG Easy Hyb buffer (Roche), and hybridization was performed overnight at 45°C.

### Detection of Q and Q-L1 by LC–MS/MS analysis

Up to 5 μg of total tRNA was digested to nucleoside level using 0.6 U nuclease P1 from *P. citrinum* (Sigma-Aldrich), 0.2 U snake venom phosphodiesterase from *C. adamanteus* (Worthington), 2 U FastAP (Thermo Fisher Scientific), 10 U benzonase (Sigma-Aldrich), 200 ng Pentostatin (Sigma-Aldrich) and 500 ng Tetrahydrouridine (Merck-Millipore) in 25 mM ammonium acetate (pH 7.5; Sigma-Aldrich) overnight at 37°C. 1 μg of total tRNA was analyzed *via* LC–MS using an Agilent 1260 series LC with a Synergi Fusion column (4 μM particle size, 80 Å pore size, 250 × 2.0 mM; Phenomenex) and an Agilent 6460 Triple Quadrupole mass spectrometer equipped with an electrospray ion source (ESI). The elution started with 100% solvent A (5 mM ammonium acetate buffer, pH 5.3) with a flow rate of 0.35 ml/min at 35°C, followed by a linear gradient to 8% solvent B (LC–MS grade acetonitrile; Honeywell) at 10 min and 40% solvent B after 20 min. Initial conditions were regenerated with 100% solvent A for 10 min. The UV signal at 254 nm was recorded *via* a multiple wavelength detector (MWD) detector at 254 nm to monitor the main nucleosides. The following ESI parameters were defined for the measurement: gas temperature 350°C, gas flow 8 l/min, nebulizer pressure 50 psi, sheath gas temperature 350°C, sheath gas flow 12 l/min, capillary voltage 3000 V, nozzle voltage 0 V. The MS was operated in the positive ion mode using Agilent MassHunter software in the dynamic MRM (multiple reaction monitoring) mode.

### Identification of preQ_1_-L1-modified RNAs by HTS

Metabolically labelled and biotin-clicked RNA was purified from total RNA or isolated small RNAs using Dynabeads™ MyOne™ Streptavidin C1 (Thermo Fisher Scientific) according to the manufacturer's instructions. Streptavidin coated magnetic beads were washed as instructed for immunoprecipitation of RNA. 20 μg of biotin-labelled RNA was incubated with the beads for 1 h at room temperature with light agitation. After washing the beads, they were resuspended in nuclease-free water, and bound RNA was dissolved from the beads by incubating the samples at 95°C for 10 min. Library preparation of immunoprecipitated RNAs for deep sequencing was done using the NEBNext Small RNA Library Prep Set for Illumina (Multiplex Compatible; New England Biolabs). 300 ng of RNA per library as starting material was used, and ligation was performed with undiluted adaptors. Adaptor-ligated cDNA was amplified with 15 cycles of PCR reaction using barcoded primers and purified using the Monarch PCR & DNA Cleanup Kit (5 μg) (New England Biolabs). Libraries were eluted in nuclease-free water, multiplexed in equimolar ratios and sequenced on one lane of the Illumina MiSeq platform using paired-end 150 bp sequencing.

### RT-qPCR quantification of tRNA^Asp^, snoR38 and snoR69

For quantification of preQ_1_-L1-labelled tRNA^Asp^, snoR38 and snoR69 from metabolically labelled and immunoprecipitated (IPed) RNAs, quantitative RT-PCR was performed using a stem-loop primer (see Supplementary Table S3). cDNA was synthesized using IPed RNAs from *S. pombe* WT and *qtr2Δ* and a sequence specific stem-loop primer. First strand synthesis was carried out using the SuperScript™ III First-Strand Synthesis System (Invitrogen) according to the manufacturer's protocol. Synthesized cDNA was subsequently used for qPCR using the PerfeCTa SYBR Green SuperMix (QuantaBio). 4 μl of cDNA was used in a reaction mix containing 12.5 μl Master Mix (Quanta, 2×) and 250 nm primers. Reactions were carried out in a total volume of 25 μl with the following thermocycling: 95°C for 2 min, followed by 40 cycles of 95°C for 10 s, 58°C for 15 s and 72°C for 20 s.

### RNA bisulfite sequencing

Bisulfite sequencing of tRNA^Asp^ was performed as previously described (38). Briefly, bisulfite-treated tRNAs were reverse transcribed using tRNA^Asp^ 3’-specific stem-loop primer followed by amplification with primers binding only to the deaminated sequence at their 5’ end. Primer sequences are listed in Supplementary Table S3. Library preparation of the PCR products was performed with the NEXTflex^R^ qRNA-Seq™ Kit v2—Set C (Bioo Scientific) according to the manufacturer's instructions and sequenced on a MiSeq platform using paired-end 150 bp sequencing. Reads were processed using in-house R scripting and the Bioconductor package ShortRead (52). Following the processing, including trimming of PCR primers, selection of high-quality reads and sorting of the reads based on the sequence in the degenerate region of the RT-primer, the reads were analyzed for bisulfite conversion using BISMA (53).

### HTS data processing

The sequencing data was adapter-trimmed using Skewer version 0.2.2 (54) and aligned to *S. pombe* non-coding RNAs (main and mitochondrial) excluding rRNA sequences from Pombase (https://www.pombase.org/) using Salmon version 14.0 (55) and HISAT2 version 2.1.1 (56), as a splice-site sensitive alignment program. The conversion of sam to bam files was performed using SAMtools (57). Aligned sequences were analyzed using custom R scripts and the Bioconductor package DESeq2 (58). Parameters were set to analyze only regions with a minimum of 10 reads and the adjusted *P*-value was set to <0.1. Additionally, independent hypothesis weighting was conducted using the Bioconductor package IHW (59,60) with an adjusted *P*-value of <0.1. Furthermore, peak calling was performed using the Bioconductor package exomePeak2 (61). Plots were generated using the integrative genomics viewer version 2.11.1 (IGV) (62).

## RESULTS

### *In vitro* incorporation of synthetic preQ_1_ analogues in prokaryotes

**Figure 1.**
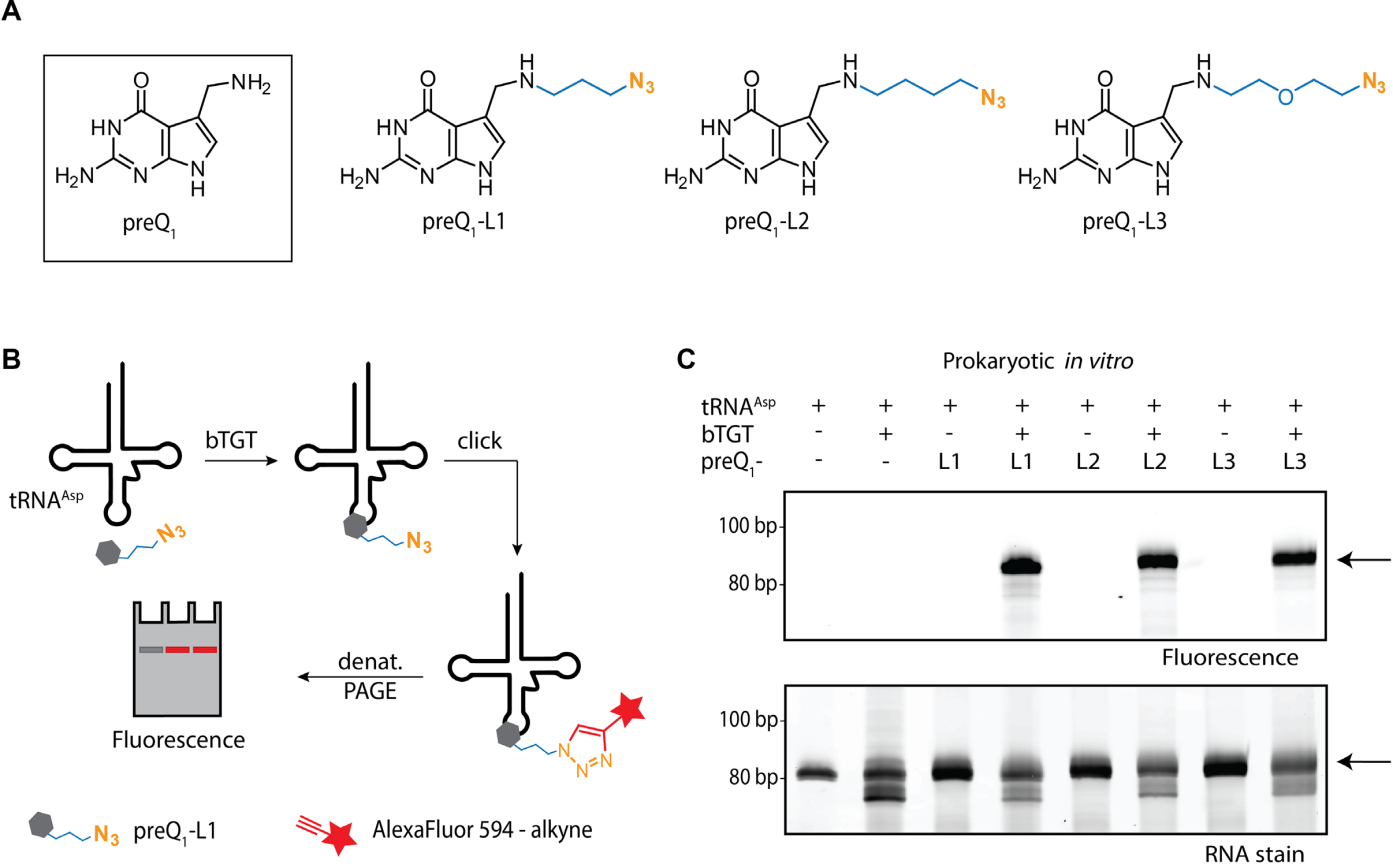
*In vitro* incorporation of preQ_1_ analogues in tRNA^Asp^. (**A**) Natural preQ_1_ and synthetic preQ_1_-ligands 1 (L1, 3-azidopropyl-preQ_1_), 2 (L2, 4-azidobutyl-preQ_1_) and 3 (L3, 2-(2-azidoethoxy)ethyl-preQ_1_) containing side chains of different length and constitution (blue) but identical terminal azide groups (orange) designated for click chemistry. (**B**) Scheme of the *in vitro* experiment: incubation of tRNA^Asp^ with preQ_1_ ligands (exemplarily shown for preQ_1_-L1) in the presence of bacterial TGT (bTGT) and subsequent click reaction with AlexaFluor 594-alkyne (red), allowing the detection of tRNA with incorporated preQ_1_ ligand *via* fluorescence scan. (**C**) Analysis of the tRNA^Asp^ click product after bTGT-catalysed incorporation of preQ_1_-ligands L1–L3 by denaturing PAGE and following visualization by fluorescence scan for AlexaFluor 594 (excitation: 532 nm, emission: 610 nm). A loading control was obtained by RNA staining with GelRed. In both scans, tRNA^Asp^ is indicated by an arrow. Untreated tRNA^Asp^ and tRNA^Asp^ incubated with bTGT or preQ_1_-ligands L1–L3, respectively, served as controls.

To assess the substrate properties of synthetic preQ_1_-ligands, their incorporation into tRNA by bacterial TGT (bTGT) was tested *in vitro* (Figure 1). For this purpose, preQ_1_-ligands 1–3 (preQ_1_-L1-3, Figure 1A), each harbouring an azide group, were incubated with tRNA^Asp^ in the presence of recombinant bTGT from *Z. mobilis*. Taking advantage of the terminal azide group, the successful *in vitro* incorporation of preQ_1_-ligands was visualized by copper(I)-catalyzed azide alkyne cycloaddition (CuAAC) click reaction of tRNA^Asp^ with the fluorescent AlexaFluor 594-alkyne in the presence of CuSO_4_, sodium ascorbate and THPTA (tris-((1-benzyl-1*H*-1,2,3-triazol-4-yl)methyl)amine) (Figure 1B and Supplementary Figure S1). As shown by fluorescence scan, all of the tested preQ_1_-ligands were incorporated to the same extent (Figure 1C), indicating that the side chains attached to the preQ_1_ structure do not hinder the recognition and turnover by bTGT. This indicates a tolerance of the bTGT active site for large ligands, similar to what was previously described for eTGT *in vitro* (42).

### *In vivo* incorporation of synthetic preQ_1_ analogues in prokaryotes

**Figure 2.**
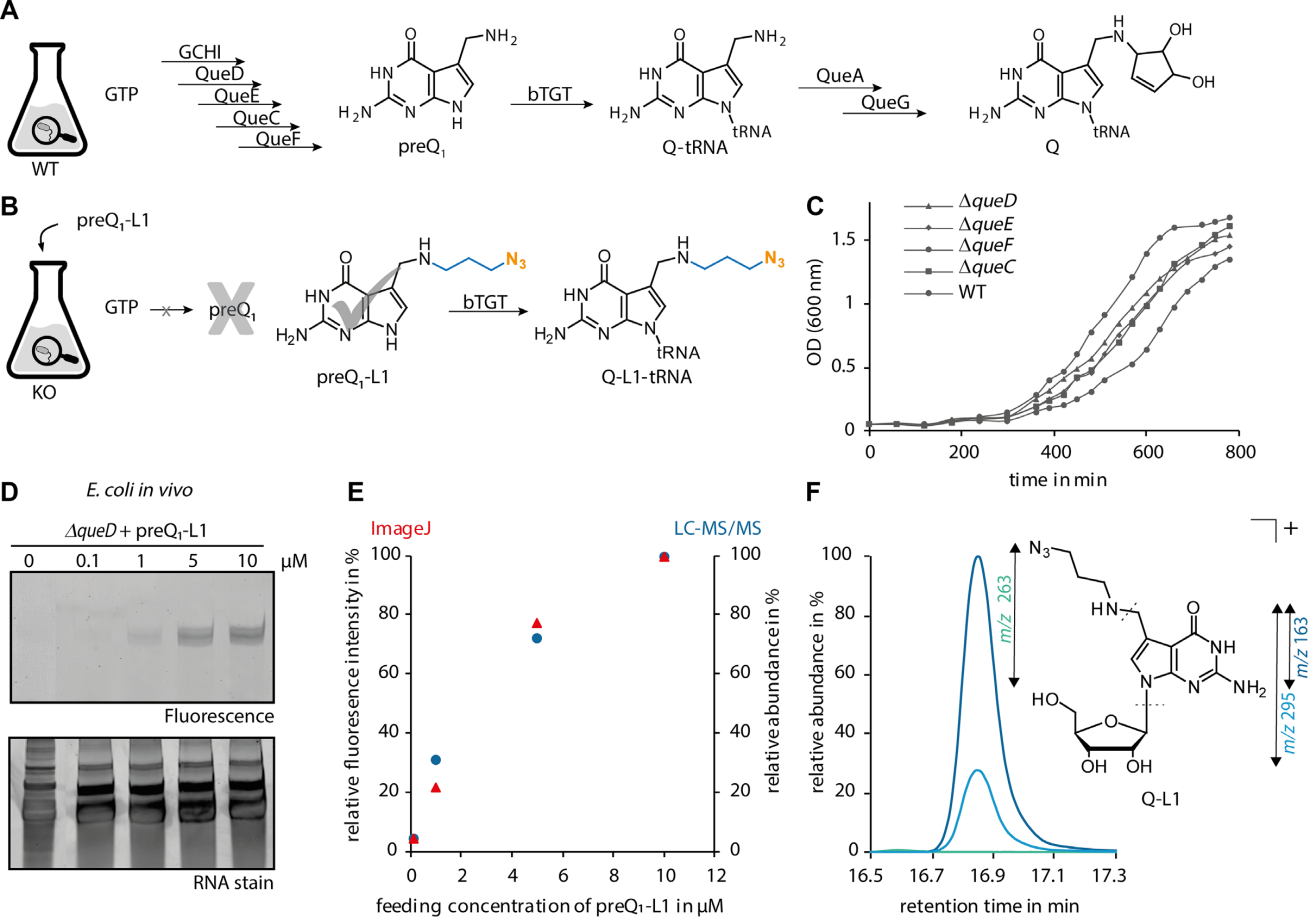
*De novo* biosynthesis of Q and induced incorporation of preQ_1_-L1 in bacteria. (**A**) Biosynthesis of Q in position 34 of tRNAs (Q34-tRNA) *via* insertion of preQ_1_ into tRNA, which is catalysed by the bacterial tRNA guanine transglycosylase (bTGT). (**B**) Treatment of *E. coli* mutant cells unable to synthesize preQ_1_ with preQ_1_-L1 and concomitant bTGT-catalysed incorporation of this analogue into tRNA. (**C**) Growth of the *E. coli* wild-type (WT) strain compared to the growth of several strains with deletions in genes encoding enzymes for Q *de novo* synthesis. (**D**) Analysis of total tRNA from *ΔqueD* cells grown with the indicated concentrations of preQ_1_-L1 after click reaction by denaturing PAGE and subsequent scanning for fluorescence of AlexaFluor 594 (excitation: 532 nm, emission: 610 nm). (**E**) Merged diagram displaying the quantification of the dose-dependent fluorescence signal obtained from (D) by ImageJ software (Wayne Rasband, NIH) (shown as red triangles) and relative quantification of Q-L1 levels by LC–MS/MS (blue dots). Peak areas of Q-L1 (*m/z* 163) were normalized to the UV signal of adenosine and set in relation to the peak area of the highest feeding concentration (10 μM). (**F**) Extracted ion chromatograms displaying the fragmentation pattern of the incorporated synthetic nucleoside Q-L1 (*m/z* 395) in LC–MS/MS experiments, normalized to the highest peak area (*m/z* 163). Product ions are assigned in the structure of Q-L1.

After the successful *in vitro* application of synthetic preQ_1_-ligands with bTGT, we proceeded to metabolic labelling of RNAs *in vivo* in *E. coli*. First experiments were performed with the smallest preQ_1_ ligand in the series, i.e. preQ_1_-L1. In a feeding experiment, where an *E. coli* wild-type (WT) strain was grown in medium supplemented with preQ_1_-L1, total tRNA was isolated and enzymatically digested to the nucleoside level for separation on an RP-C18 HPLC column and subsequent analysis of the Q levels by MS/MS. Of note, queuosine exhibits a fragmentation pattern differing from the standard nucleosides. Instead of the exclusive fragmentation at the *N*-glycosidic bond, cleavage of the ribose in combination with cleavage of the amino linker with a mass shift *m/z* 410 to *m/z* 163 was determined as the most abundant product ion (Supplementary Figure S2a) eluting at a retention time of 12.2 min in the WT sample. Using a fragmentation pattern for the incorporated synthetic nucleoside (Q-L1) that was inferred from that of native queuosine, additional signals for the expected transitions were detected at 16.9 min (Figure 2). Since the product ion *m/z* 163 was the most prevalent species, it was chosen as diagnostic ion in subsequent LC–MS/MS experiments. Monitoring this product ion produced a strong signal for queuosine and only a weak signal for Q-L1 (Supplementary Figure S3c). We concluded that preQ_1_-L1 was indeed incorporated, but also that it was a weak competitor against the endogenous bacterial preQ_1._ Consequently, we reasoned that abrogating preQ_1_ biosynthesis would facilitate the incorporation of the supplied preQ_1_-ligands. Considering the various steps of Q *de novo* synthesis in *E. coli* (Figure 2A), four different gene deletions, namely *ΔqueD*, *ΔqueE*, *ΔqueC* and *ΔqueF*, were tested for generation of preQ_1_ by monitoring the presence of Q at position 34 of tRNAs. The deletion strains showed no significant growth defects compared to the wild type (Figure 2C). To validate the absence of Q *de novo* biosynthesis, tRNA was isolated and analyzed by LC–MS as before; none of the deletion strains generated measurable levels of Q (Supplementary Figure S2b–e). Since all deletions were on a par regarding growth and absence of Q in the isolated total tRNA, *ΔqueD* was chosen for further experiments, given that the absence of QueD prevents *de novo* synthesis of Q in its earliest stages and avoids synthesis of any precursor form (e.g. preQ_0_ in *ΔqueF*) that was reported to be incorporated by the TGT and might thus compete with preQ_1_-L1 (Supplementary Figure S3c) (13).

To determine a suitable feeding concentration, the *ΔqueD* cells were supplemented with increasing amounts of preQ_1_-L1 (Figure 2B), which did not impair the bacterial growth compared to the control without preQ_1_-L1 feeding (Supplementary Figure S3b). Total tRNA isolated from thus treated *ΔqueD* cells was labelled *via* CuAAC click chemistry and subsequently analysed by denaturing urea PAGE. The fluorescence scan revealed clearly visible fluorescent bands after feeding with preQ_1_-L1 in a dose-dependent manner, providing evidence for the enzymatic incorporation of the analogue into *E. coli* tRNA *in vivo* (Figure 2D). The signal intensity was quantified using ImageJ software and plotted against the feeding concentration of preQ_1_-L1 (Figure 2E). To validate these observations, total tRNA isolated from the *ΔqueD* strain treated with preQ_1_-L1 was further analysed *via* LC–MS/MS. The MS-based analysis of total tRNA isolated from the *ΔqueD* fed with increasing concentrations of preQ_1_-L1 (0.1–10 μM) confirmed the results obtained from denaturing urea PAGE analysis and related quantification of the fluorescence signal (Figure 2E). Both plots indicate a beginning saturation around 10 μM. Based on the above, a ligand concentration of 5 μM for further feeding experiments was identified as a viable compromise between ligand material consumption and labelling efficiency. Supplementation of a *Δtgt* strain with the optimized concentration of 5 μM preQ_1_-L1 resulted in no signal for Q-L1 in LC–MS/MS measurements, thus confirming that the incorporation was catalysed by bTGT (Supplementary Figure S3c).

Since preQ_1_-L1 was successfully incorporated into RNA in bacteria, the *in vivo* experiments were expanded to preQ_1_-L2 and preQ_1_-L3. However, in contrast to the previously described *in vitro* experiments, neither feeding preQ_1_-L2 nor preQ_1_-L3 at the optimized concentration of 5 μM or at higher concentrations (10 μM for preQ_1_-L2 and 20 μM preQ_1_-L3) led to a clear fluorescence signal in the clicked total tRNA samples (Supplementary Figure S3a), indicating that the incorporation efficiency of preQ_1_-L2 and preQ_1_-L3 into tRNA *in vivo* was drastically lower compared to preQ_1_-L1. Since the *in vitro* results indicate indifference of the TGT enzyme towards the alkyl-modified preQ_1_-ligands, the low incorporation *in vivo* suggests lower bioavailability of preQ_1_-L2 and preQ_1_-L3 for the bacteria.

### *In vivo* interactions of synthetic preQ_1_ analogues in prokaryotes

To investigate possible changes in the ensemble of molecular interactions undergone by Q-L1-carrying tRNA under physiological conditions, we turned to the analysis of polysomes. Given that these consist of actively translating ribosomes, their components, including tRNA, can be considered functional in interactions with essential molecular factors involved in translation. We thus aimed at determining the ratio of Q-L1-carrying tRNAs from polysomes versus that in the remainder of tRNAs.

**Figure 3.**
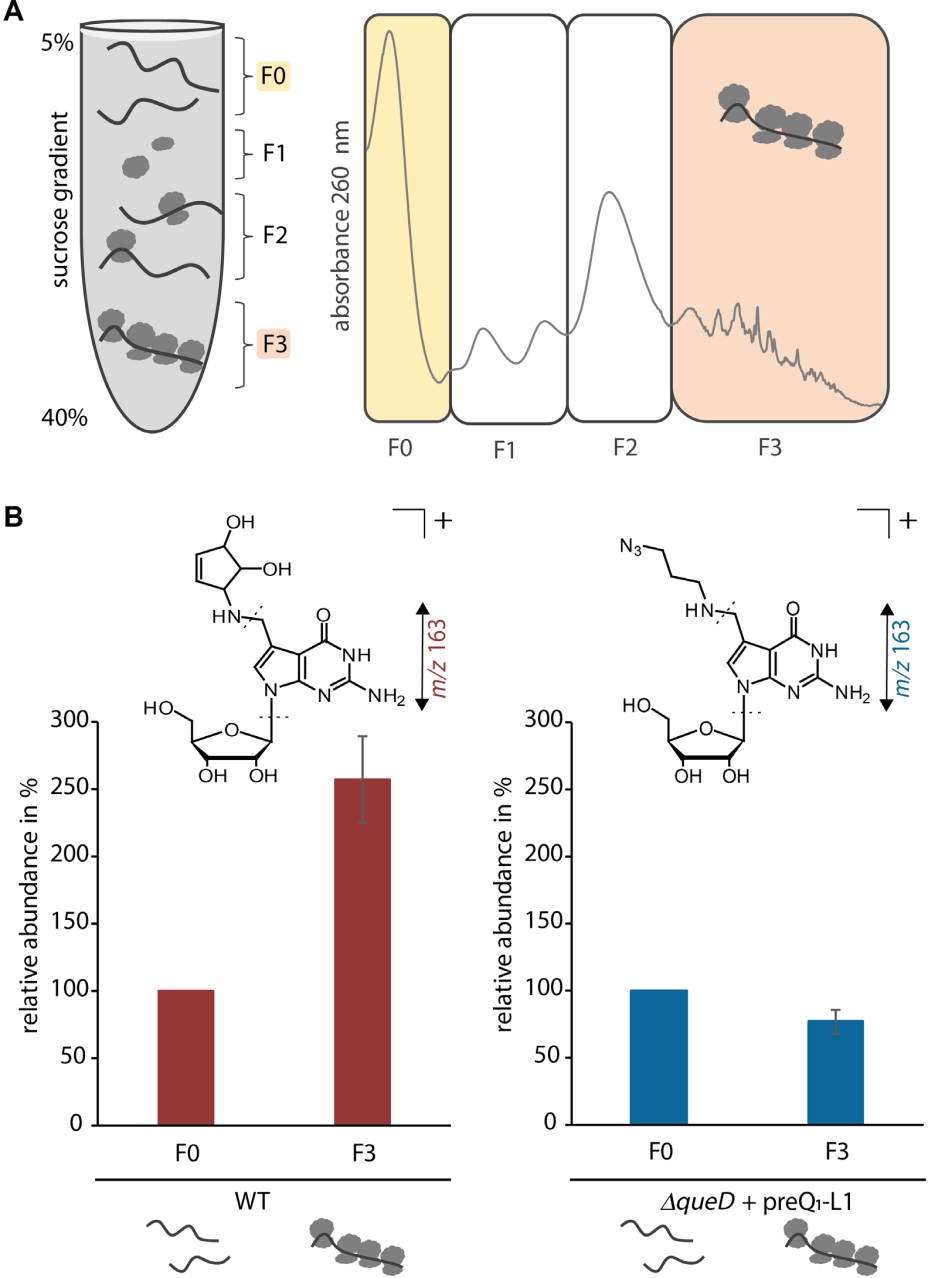
*E. coli* polysome preparation and analysis of isolated tRNA obtained from these samples by LC–MS/MS. (**A**) Schematic distribution of fractions F0-F3 from a cell lysate after sucrose gradient (5–40 %) fractionation and ultracentrifugation and representative UV trace at 260 nm (representing RNA) across the sucrose gradient. (**B**) Relative quantification of Q (*m/z* 410 → 163, red) and Q-L1 (*m/z* 395 → 163, blue) in tRNA purified from fractions F0 and F3 of WT and *ΔqueD* cells supplemented with 10 μM preQ_1_-L1 *via* LC–MS/MS. Peak areas were normalized to the UV signal of adenosine and related pairwise to the respective F0 fraction which was set to 100%. The average of normalized and related fractions F0 and F3 of three independent biological replicates are shown.

For this purpose, cell lysates from *E. coli* WT and *ΔqueD* cells supplemented with 10 μM preQ_1_-L1 were applied to a sucrose gradient (5–40%), enabling the separation of different fractions according to their size after ultracentrifugation. As schematically shown in Figure 3A, free RNAs including tRNAs and some mRNAs were located in fraction F0 at the top of the gradient (5% sucrose), while polysomes accumulated in fraction F3 at a sucrose concentration of ∼40%. This separation was monitored by UV absorbance at 260 nm, and the different fractions were collected. Subsequent to fractionation, total RNA was extracted from these fractions and applied to denaturing PAGE for purification of tRNA *via* gel elution. Digested tRNA samples were subjected to LC–MS/MS analysis, and the respective abundances of Q and Q-L1 were compared between the free RNA fraction F0 and the polysomal fraction F3 (Figure 3B). Interestingly, in WT cells, endogenous Q was more abundant in tRNAs isolated from the polysomal fraction compared to fraction F0. This suggests that queuosinylated tRNAs are selectively enriched in polysomes that are actively engaged in translation.

In contrast, the Q-L1 level in polysomal tRNA (F3 fraction) from preQ_1_-L1 fed *ΔqueD* cells reached a similar amount compared to its level in the respective F0 fraction. This may reflect either a deficit in the aforementioned selection, or a cumulation of minor detrimental effects at the different steps of translation. However, the data clearly illustrate that Q-L1-containing tRNAs actively engage in protein biosynthesis and are able to sustain it at a high enough level to not cause any perceivable growth phenotype.

### *In vitro* incorporation of synthetic preQ_1_ analogues in eukaryotes

In a next step, the investigations were extended from bacteria to eukaryotes. Of note, eukaryotes do not possess the enzymes to synthesize queuosine *de novo*, but salvage it from external sources for incorporation into tRNA (16). *S. pombe* is a particularly well-suited single cell eukaryotic model organism, because salient features of queuosine have already been elaborated in this yeast, and queuosine levels can easily be manipulated by supplementation of the growth medium with queuine (38).

**Figure 4.**
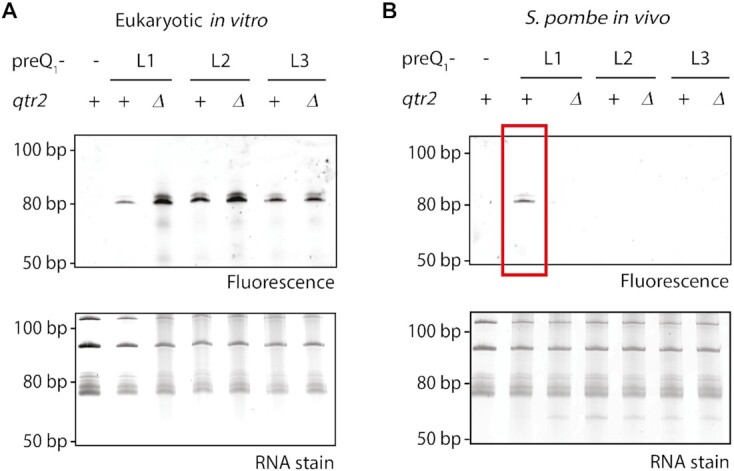
*In vitro* and *in vivo* incorporation of preQ_1_-ligands in *S. pombe* tRNA. (**A**) Analysis of the total RNA click product after human tRNA guanine transglycosylase (hTGT)-catalysed incorporation of preQ_1_-ligands L1–L3 into RNA from *S. pombe* by denaturing PAGE and visualization by fluorescence scan for AlexaFluor 594 (excitation: 532 nm, emission: 610 nm). Total RNA was extracted from *S. pombe* WT cells containing functional TGT (+) and *qtr2Δ* cells that lack functional TGT (*Δ*), which were both cultured in the presence of queuine. The incubation of total RNA from WT cells without preQ_1_-ligand (-) served as a negative control. A loading control was obtained by RNA staining with SybrGold. (**B**) Analysis of total tRNA from *S. pombe* WT (+) and *qtr2Δ* (*Δ*) cells that were cultured in the presence of 0.1 μM of the respective preQ_1_-ligand after click reaction by denaturing PAGE and subsequent visualization as described above. Total RNA from *S. pombe* WT cells supplemented with 0.1 μM queuine (–) instead of preQ_1_-ligands was used as a negative control.

We next tested the ability of eTGT to incorporate the preQ_1_-ligands into RNA *in vitro*. As substrates for this reaction, total RNA was isolated from *S. pombe* wild-type cells or *qtr2Δ* cells cultured in the presence of queuine. In WT cells, this results in Q-modification of the tRNAs, whereas *qtr2Δ* cells lack the essential Qtr2 subunit of *S. pombe* eTGT, therefore maintaining a guanosine in position 34 of the respective tRNAs. Total RNA preparations of these strains were incubated with the preQ_1_ ligands in presence of hTGT, and subsequently labelled by click reaction. The incorporation of all three ligands into tRNA from both *S. pombe* strains was measured by fluorescence scan (Figure 4A and Supplementary Figure S4b). In comparison to the fluorescence signals of the tRNA from WT cells, the respective signals of the *qtr2Δ* tRNAs showed significantly higher intensities. This indicates that more tRNAs unmodified at position G34 are available for *in vitro* modification with preQ_1_-L1 in the *qtr2Δ* sample. In contrast, in WT cells only guanosines that were not replaced by Q despite the presence of a functional enzyme remained for the *in vitro* reaction. Unlike observed for the bTGT, the hTGT incorporated the preQ_1_-ligands to differing degrees, indicating a higher ability to distinguish between these analogues in accordance with previously published results by Kelly and co-workers (41). Additionally, incubation of *in vitro* transcribed tRNAs Asp, His, Tyr and Asn with human TGT and preQ_1_-L1 showed successful incorporation of the analogue into all of the four tRNAs (Supplementary Figure S4a).

### *In vivo* incorporation of synthetic preQ_1_ analogues in eukaryotes

Subsequent to the successful *in vitro* experiment, the *in vivo* incorporation of the synthetic preQ_1_ analogues was examined in *S. pombe*. To this end, *S. pombe* WT and *qtr2Δ* cells (as a control), were cultured in the presence of preQ_1_-ligands in medium that otherwise lacked Q or q, and RNA was isolated and analysed as before. After click reaction, a fluorescence signal was detected in the RNA isolated from the WT cells treated with preQ_1_-L1, but not *qtr2Δ* (Figure 4B), showing that the presence of Q-L1 in tRNA *in vivo* depended on functional TGT. As in bacteria, preQ_1_-L1 did not negatively affect cell growth (Supplementary Figure S4c), and no labelling was observed with preQ_1_-L2 and -L3, again indicating that their derivatives are not bioavailable for incorporation into tRNAs *in vivo*.

Collectively, the above experiments indicate that preQ_1_-L1 can readily be employed as a proxy for Q from the perspective of synthetic biology. To further develop this compound for the investigation of the epitranscriptome, we made use of the click chemistry feature of preQ_1_-L1 to identify RNAs into which it was incorporated *in vivo* by eTGT.

**Figure 5.**
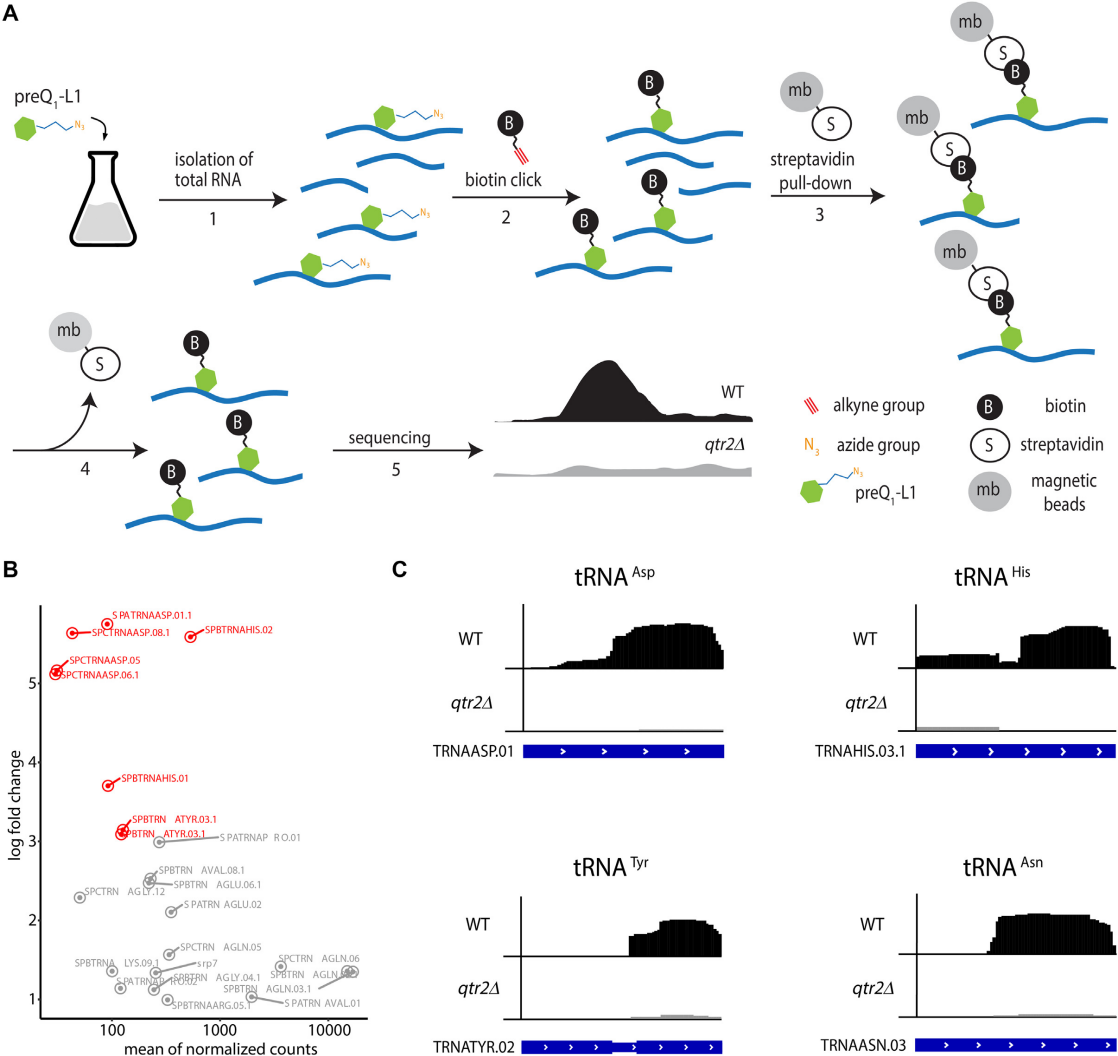
*In vivo* identification of Q-modified RNAs in *S. pombe* based on metabolic labelling with preQ_1_-L1 and high-throughput sequencing (Q-RIP-Seq). (**A**) Concept of metabolic labelling and immunoprecipitation of Q-modified RNAs. *S. pombe* was cultured in the presence of 0.1 μM preQ_1_-L1, leading to incorporation into otherwise Q-modified RNAs. Total RNA was extracted (1) and bio-conjugated *in vitro* with alkyne-functionalized biotin (2). Biotin-labelled RNAs were subsequently affinity-purified using streptavidin-coated magnetic beads (3), reverse-transcribed and subjected to high-throughput sequencing (5). As a control, metabolic labelling was performed in an *S. pombe* strain lacking TGT (*qtr2Δ*). (**B**) Log2 fold change of normalized read counts of RNAs from WT compared to *qtr2Δ* determined by exomePeak2. Red: tRNA^Asp^, tRNA^His^ and tRNA^Tyr^; (three independent replicates). (**C**) Q-RIP-Seq of tRNA^Asp^, tRNA^His^, tRNA^Tyr^ and tRNA^Asn^ after metabolic labelling with preQ_1_-L1 in *S. pombe* WT and *qtr2Δ* cells. Coverage of the tRNA sequences from modified (WT, black) and unmodified *(qtr2Δ*, grey) samples is shown. The transcript architecture is shown below with thin and thick parts representing introns and mature tRNA sequences. Replicate 1 of three independent experiments is shown. Plots were generated using IGV.

For this purpose, total RNA isolated from *S. pombe* wild-type or *qtr2Δ* cells that were cultured in the presence of preQ_1_-L1 was bio-conjugated *in vitro* with alkyne-functionalized biotin. Subsequent to affinity purification using streptavidin-coated magnetic beads, the biotin-labelled RNA was subjected to reverse transcription and high-throughput sequencing (Figure 5A, termed Q-RIP-Seq). The analysis showed that the known cytosolic Q-tRNAs tRNA^Asn^, tRNA^Asp^, tRNA^His^ and tRNA^Tyr^ were significantly enriched from WT, but not *qtr2Δ* cells (*n* = 3, *P*_adj_ < 0.1, Figure 5B, C and Supplementary Figure S5). Other enriched signals from snoR38 and snoR69 were scrutinised as potential substrates of TGT-mediated incorporation of preQ_1_-L1. However, neither APB Northern blotting nor quantification by q-RT-PCR substantiated this hypothesis (Supplementary Figure S6). Interestingly, mitochondrial tRNA^Asn^, when analysed for q content by APB-northern blot, was queuosinylated to about 50% (Supplementary Figure S5b). The fact that no mitochondrial tRNA sequences were found in Q-RIP-Seq could mean that they are too low in abundance. An alternative explanation would be that preQ_1_-L1 is not incorporated into mitochondrial tRNA. The above findings indicate that the four known Q-tRNAs are the only cytosolic RNAs that are Q-modified in *S. pombe*, which is congruent with crosslinking-based studies in human cells (41). These results establish that any major metabolic influence resulting from feeding preQ_1_-L1 would be mediated through the four classical tRNA substrates of TGT. It should, however, be noted that an early study reported preQ1-modification *in vitro* of larger RNA species in *E. coli* (63).

### *In vivo* interactions of synthetic preQ_1_ analogues in eukaryotes

**Figure 6.**
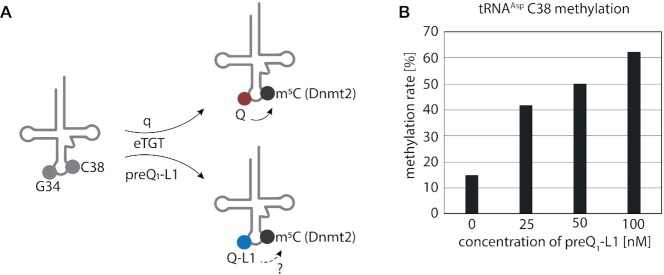
Incorporation of preQ_1_-L1 in tRNA^Asp^ in *S. pombe* stimulates C38 methylation by Dnmt2. (**A**) Incorporation of q or preQ_1_-L1 by eukaryotic tRNA guanine transglycosylase (eTGT) affects Dnmt2 activity in *S. pombe* (Pmt1). (**B**) Determination of tRNA^Asp^ methylation levels at C38 in total RNA from *S. pombe* WT supplemented with the indicated concentrations of preQ_1_-L1 by RNA bisulfite sequencing combined with high throughput sequencing.

Having established that preQ_1_-L1 is actively incorporated into native tRNAs, we next investigated a particularly interesting effect of queuosine, namely a so-called tRNA modification circuit, where the formation of one modification is enhanced by the presence of another modification (64). The particular circuit involving queuosine was first identified in *S. pombe*. We had shown earlier by RNA bisulfite sequencing that the formation of m^5^C38 in tRNA^Asp^ by the Dnmt2 tRNA methyltransferase is strongly enhanced by the presence of queuosine at position 34 (Figure 6A) (38,49).

We therefore asked whether Q-L1 can serve as a biologically active surrogate for queuosine in this circuit. Figure 6B shows the m^5^C38 levels of tRNA^Asp^ in response to increasing concentrations of preQ_1_-L1 in medium otherwise free of queuosine derivatives. A clear dose-dependent increase of the C38 methylation level was observed, indicating that the incorporated preQ_1_-L1 is functionally integrated into this modification circuit, efficiently replacing queuosine in its capability of triggering Dnmt2 activity in *S. pombe*. Considering the direct functional connection of Q/Q-L1 and m^5^C38, the increase of the C38 methylation level from 15% in non-treated culture up to 60% in cultures supplemented with 100 nm preQ_1_-L1 points to its incorporation in significant amounts in *S. pombe*. However, it is important to mention that the effect of preQ_1_-L1 incorporation on tRNA^Asp^ methylation is less efficient compared to the known effect of Q under normal conditions, as we previously reported (38). The incorporation efficiency in *E. coli* can only be gauged even more indirectly, namely by comparison of fluorescent signals after click (Supplementary Figure S4d).

Lastly, we were also able to demonstrate successful incorporation of preQ_1_-L1 in HeLa human cells deprived of q (Supplementary Figure S7a). In analogy to the earlier presented analysis of *E. coli* polysomes, we also investigated the levels of Q-L1 in tRNA purified from F0 and F3 of accordingly treated HeLa cultures. Similar to our observations in *E. coli*, the amount of Q-L1 detected in the polysomal tRNA (F3 fraction) from preQ_1_-L1 fed HeLa cells was comparable to its level in the respective F0 fraction, indicating that Q-L1-containing tRNAs actively engage in protein biosynthesis *in cellulo* (Supplementary Figure S7b). This result indicates relevance of our investigations with respect to biomedical considerations, e.g. potential therapeutic interventions.

## DISCUSSION

Interest in concepts for the incorporation of modified and/or non-natural derivatives of metabolites into nucleic acids has been steadily increasing, boosted in part by a surge in RNA modification research, and, more recently, in mRNA-based vaccines. Post-synthetic derivatization of RNA *in vitro*, e.g. by methyltransferases has been exploited for labelling in conjunction with click chemistry (65–69). In the queuosine field, a number of q-derived compounds, including clickable tetrazine derivatives, have been incorporated into native RNA preparation *in vitro* using recombinant TGT, and applied to fluorescent labelling, affinity purification, and interactome research (42–44,70,71). In a previous study, Brooks *et al.* reported that azide congeners of preQ_1_ lacking the methylene amine were not incorporated by the TGT which they traced to the necessity of this structural element for a successful binding to the enzyme forming hydrogen bonds between aminoacid residues Leu231 and Met260 of the enzyme (72,73). Although, as mentioned, strong indirect evidence (34) suggested that incorporation of nonnatural q derivatives should be feasible in principle, no *in vivo* labelling of Q-tRNAs with clickable q-derivatives has been demonstrated so far.

Overall, concepts and applications in the RNA field currently move from *in vitro* (74) to metabolic feeding approaches *in cellulo* and *in vivo*. Here, the use of noncanonical nucleoside structures has opened up new experimental avenues in the community. As an example, in RNA modification research feeding of methionine analogues featuring e.g. propargyl residues, has enabled their incorporation into RNA *in lieu* of methyl groups. Subsequent derivatization by click chemistry was exploited for determination of modification sites (75,76,66).

An important progress featured in our work is that we demonstrate low toxicity of the labelling compound and provide corresponding data at the molecular level. Elsewhere in the field, little attention is paid to the physiological impact of surrogate feeding. In most cases, a moderate survival rate in cell culture is sufficient to conduct e.g. -omics type analyses after incorporation (75–77). However, in the next steps of its development, the field might conceivably move to applications in model organisms. Here, by the latest, one will need to adopt concepts from medicinal chemistry, such as cell permeability, and toxicity. In this respect, the work presented here pioneers the combination of metabolic feeding of clickable surrogates with investigations into their physiological molecular impact after cellular uptake and their usage for the enrichment and identification of RNA species that were labelled *in vivo* by endogenous TGT. Apart from the observation of growth inhibition of q derivatives in eukaryotic cell culture, which are somewhat suggestive (78), there is strong indirect evidence for the actual incorporation of a q-derivative by TGT *in vivo* in mouse (34), however without direct analysis of the tRNA. Significantly, said case features a background of medicinal chemistry, and the compound used is structurally related to our preQ1-L series used here. It does, however, feature a lipophilic phenylpropyl sidechain which is likely causative of, or enhancing the compound's cell permeability and biodistribution.

In the present work, we have developed the azido-propyl-derivative preQ_1_-L1 as a bioactive surrogate for preQ_1_*in vivo*. preQ_1_-L1 is taken up into unicellular prokaryotes as well as into eukaryotes, and incorporated into the known tRNA substrates of TGT. The resulting nucleoside is semi-synthetic in that its sugar moiety is native, while its nucleobase is synthetic. Its azide moiety can be employed to metabolically label and isolate Q-modified RNAs by affinity purification after conjugation by click chemistry. We used this feature to confirm similar data from human cells, obtained after UV-crosslinking (41). Taken together, this means that the single most important molecular interaction for a physiological impact of q (or preQ_1_-L1) is mediated through position 34 in the anticodons of the four known TGT substrate tRNAs.

Known molecular interactions issuing from this nucleobase are mostly restricted to tRNA aminoacylation and mRNA decoding, which we have interrogated by investigating the amount of Q-L1 carrying tRNAs on polysomes. While Q-L1 was less abundant there than was native Q, it was clearly present, featuring an equal distribution between actively translating tRNAs and the cytoplasmic pool in both bacterial and human cell preparations.

One other known effect of Q was also faithfully emulated by Q-L1, namely the stimulation of m^5^C38 formation by Dnmt2 in the anticodon stem of tRNA^Asp^, representing a so-called modification loop. Technically speaking, we report the first-ever manipulation of a modification loop by atomic mutagenesis *in vivo*.

In spite of numerous described Q-dependent implications in various diseases, starting from cancer (29–32) to neurological and neuropsychiatric disorders, such as multiple sclerosis, schizophrenia and Parkinson (79,80,33,34), a defined mechanism explaining the role of Q in these pathologies is still missing (28). Recently, we discovered a direct connection between Q, accuracy and the speed of codon-biased translation (27,28), which promotes protein folding and prevents the accumulation of misfolded proteins. The fact that Q-L1 is functionally involved in the translational process in a ‘minimally invasive’ system, opens the possibility to study the roles of Q34 modifications in protein translation in normal and pathogenic human cell lines, directly combining click chemistry or LC–MS/MS with polysome profiling.

In summary, the combination of very few queuosinylation sites and the effective functional replacement of Q by Q-L1 on the molecular level, makes the q/Q system uniquely suited for a ‘minimally invasive’ placement of a non-natural nucleobase within the total cellular RNA.

## DATA AVAILABILITY

HTS data for Q-RIP-Seq experiments are available in the NCBI GEO database, record GSE210404. All data needed to evaluate the conclusions in the paper are present in the paper and/or Supplementary Data. Additional data related to this paper may be requested from the authors.

## Supplementary Material

gkac822_Supplemental_File
