# Supplementary material for: Functional integration of a semi-synthetic azido-queuosine derivative into translation and a tRNA modification circuit
Source: Nucleic Acids Res. 2022 Sep 28;50(18):10785–800. doi: 10.1093/nar/gkac822 (PMC9561289; doi:10.1093/nar/gkac822)
Supplement: gkac822_Supplemental_File [file gkac822_supplemental_file.docx]

**Supplementary Data**

**Functional integration of a semi-synthetic azido-queuosine derivative into translation and a tRNA modification circuit**

Larissa Bessler^1,†^, Navpreet Kaur^2,†^, Lea-Marie Vogt^1,†^, Laurin Flemmich^3^, Carmen Siebenaller^4^, Marie-Luise Winz^1^, Francesca Tuorto^5^, Ronald Micura^3^, Ann E. Ehrenhofer-Murray^2,^* and Mark Helm^1,^*

^1^Institute of Pharmaceutical and Biomedical Sciences, Johannes Gutenberg-University Mainz, 55128 Mainz, Germany
^2^Institute of Biology, Humboldt-Universität zu Berlin, 10117 Berlin, Germany
^3^Department of Organic Chemistry, University of Innsbruck, 6020 Innsbruck, Austria
^4^Department of Chemistry – Biochemistry, Johannes Gutenberg-University Mainz, 55128 Mainz, Germany.
^5^Division of Biochemistry, Mannheim Institute for Innate Immunoscience (MI3), Medical Faculty Mannheim, Heidelberg University, Mannheim, Germany

* To whom correspondence should be addressed. Tel: +49 (0) 6131 39 25731; Fax: +49 (0) 6131 39 20373; Email: [mhelm@uni-mainz.de](mailto:mhelm@uni-mainz.de)
Correspondence can also be addressed to Ann E. Ehrenhofer-Murray. Tel: +49 (0) 30 2093 49630; Fax: +49 (0) 30 2093 49641; Email: [ann.ehrenhofer-murray@hu-berlin.de](mailto:ann.ehrenhofer-murray@hu-berlin.de)

^†^ Joint Authors

**Supplementary Table S1:** *S. pombe* strains used in this study.

| Designation | Genotype | Source |
| --- | --- | --- |
| AEP1 | *h^-^ leu1-32 ura4-D18 his3-D3* | YGRC |
| AEP288 | *h^-^ leu1-32 ura4-D18 his3-D3* *qtr2*Δ::*NatMX* | (1) |

**Supplementary Table S2:** Plasmids used in this study.

| Designation | Genotype | Source |
| --- | --- | --- |
| pAE1688 | pJET1-tRNA^Asp^ (*S.pombe*) | (2) |
| pAE2975 | pASK-IBA13Plus - *Z. mobilis* TGTStrep-tag® II N‑terminal | (3) |
| pAE2963 | pCDF-Duet - hQTRT1-6xHis & hQTRT2 | (4) |

**Supplementary Table S3:** Oligonucleotides used in this study.

| Designation | Sequence | Purpose |
| --- | --- | --- |
| 5S rRNA | 5‘-biotin- ACCCCGGATTCCCATGTTGTCTCCAACCATAGTAC-3‘ | rRNA depletion |
| 5.8S rRNA | 5‘-biotin- CGTTCTTCATCGATGCGAGAGCCAAGAGATCCGTT-3‘ | rRNA depletion |
| tRNA Asp RT-Primer | 5’-CTCaactggattggctnnnnngataaatccagttgagtggCTCTCCCT -3‘ ^a^ | RT |
| tRNA Asp bisulfite.fwd | 5’- TTAGTATAGGGGTAGTATAT-3’ | Bisulfite sequencing |
| stemloop.rev | 5‘- CGATCANNNNCTCAACTGGATTGGCT -3‘ ^b^ | Bisulfite sequencing |
| tRNAAsp_probe | 5’-biotin-GGGCTGCAAGCGTGACAGG-3’ | Northern |
| snoR38_probe | 5‘-biotin-CTCAACACTATGCTTTAGACAGGG-3‘ | Northern |
| snoR69_probe | 5‘-biotin-GCGTACTCGTCAATGTAAATAC-3‘ | Northern |
| RT-Primer_tRNAAsp | 5’-AATCACTCAACTGGATTGGCT  nnnnnGATAAATCCAGTTGAGTGGCTCTCCCTC-3’ ^a^ | RT |
| RT-Primer_sno38 | 5’-AATCACTCAACTGGATTGGCT  nnnnnGATAAATCCAGTTGAGTGGCAGATTTAC-3’ ^a^ | RT |
| RT-Primer_snoR69 | 5’-AATCACTCAACTGGATTGGCT  nnnnnGATAAATCCAGTTGAGTGGGTTCAGATA-3’ ^a^ | RT |
| qPCR_tRNAAsp_fwd | 5’-ATAGGGGTAGTACACAAGCCTGT-3’ | qPCR |
| qPCR_sno38_fwd | 5’-ACAGTTATCCCTGTCTAAAGCATAG-3’ | qPCR |
| qPCR_snoR69_fwd | 5‘-CTTCGTTAAACCCAGCTCAC-3’ | qPCR |
| qPCR_rev | 5’-CGATCAATCACTCAACTGGATTGGCT-3’ | qPCR |

^a^ Nucleotides marked as “n” are random nucleotides used as indices
^b^ Nucleotides marked as “N” are the barcode region

**Supplementary Table S4:** Software used in this study.

| Software | Version |
| --- | --- |
| R | 3.6.3 |
| Skewer | 0.2.2 |
| Salmon | 14.0 |
| HISAT2 | 2.2.1 |
| SAMtools | 1.12 |
| DESeq2 | 1.26.0 |
| ExomePeak2 | 2.16.0 |
| IGV | 2.11.1 |
| M6A viewer | 1.6.1 |


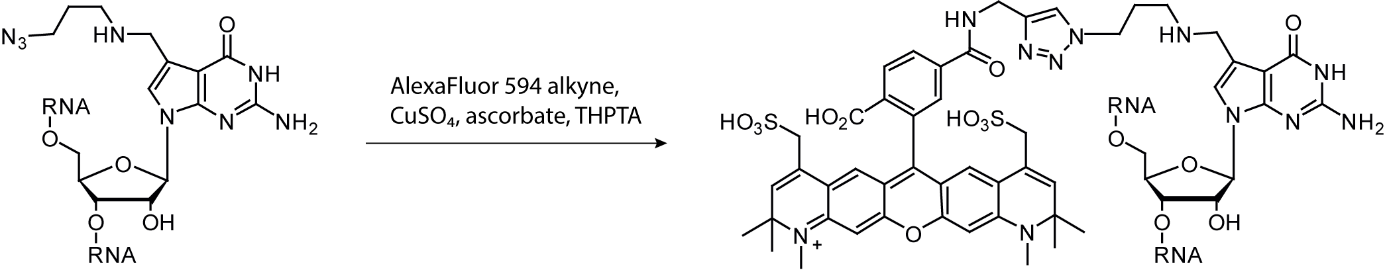


**Supplementary Figure 1: Click derivatization of preQ_1_-L1-containing RNA by a fluorescent alkyne.** THPTA = Tris((1-hydroxy-propyl-1H-1,2,3-triazol-4-yl)methyl)amine.


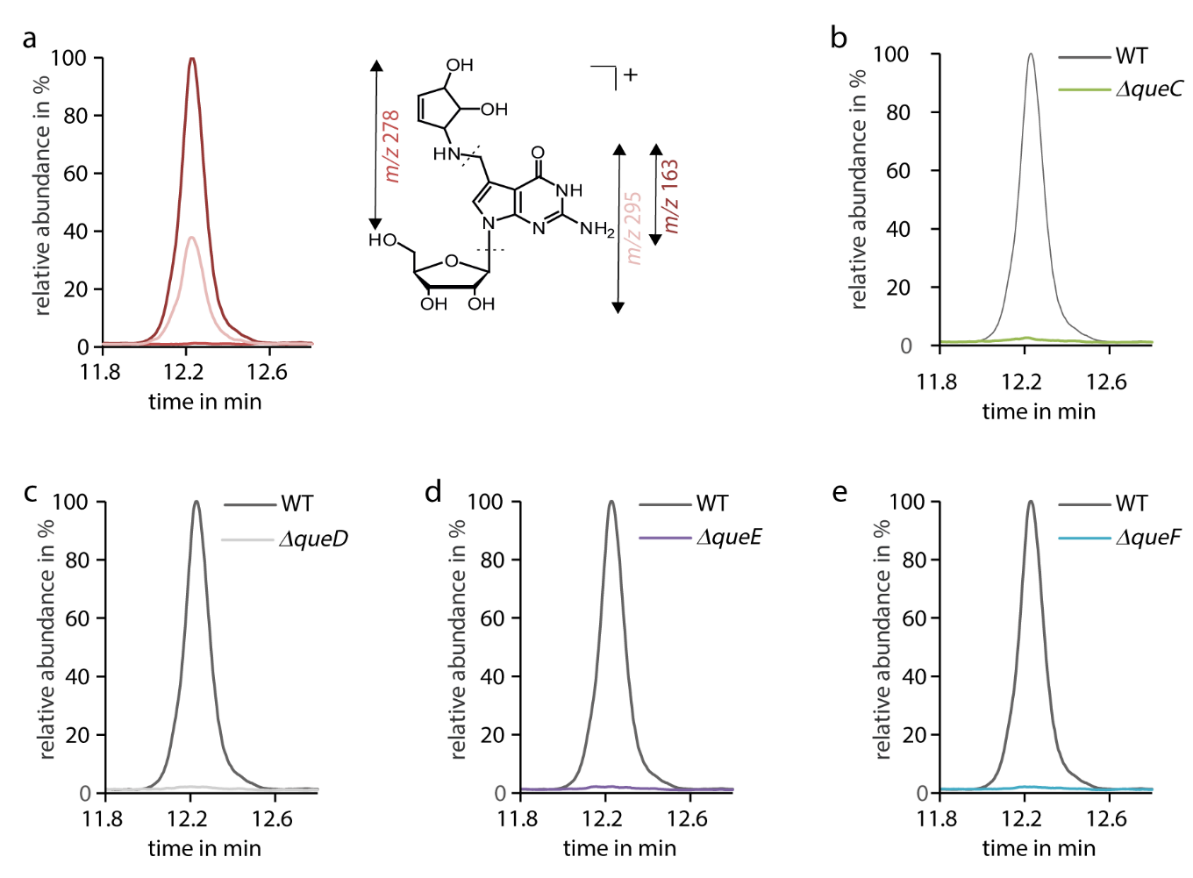


**Supplementary Figure 2: LC-MS analysis of queuosine levels.** **a** Extracted ion chromatograms displaying the fragmentation pattern of Q (*m/z* 410) in LC-MS/MS experiments, normalized to the highest peak area (*m/z* 163). Product ions are assigned in the structure of Q. **b** Relative LC-MS/MS quantification of Q levels in digested total tRNA from wild type and *ΔqueC* mutant cells, normalized to the UV signal of adenosine and set in relation to the peak area of Q in the WT cells. **c**, **d** and **e** show similar analysis of total tRNA from wild type and *ΔqueD, ΔqueE* and *ΔqueF*, respectively.

**
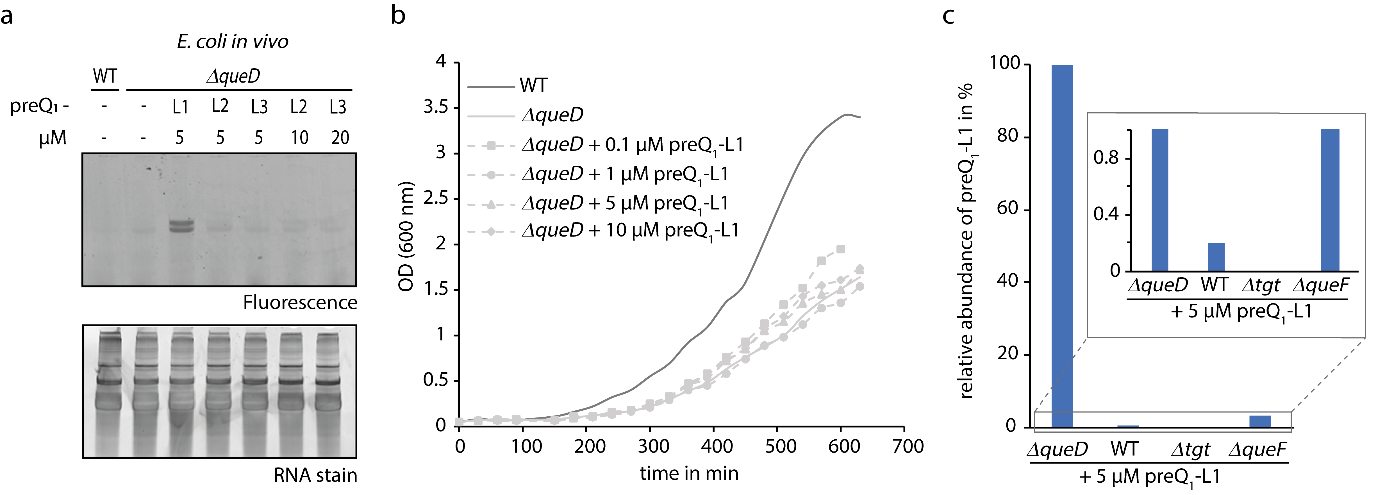
**

**Supplementary Figure 3: Detection of incorporation of preQ_1_-ligands L1-3 into total tRNA isolated from a *ΔqueD E. coli* strain via click derivatization.** **a** Analysis of total tRNA from *ΔqueD* grown with the indicated concentrations of preQ_1_-ligands L1-3 after click reaction by denaturing PAGE and subsequent scanning for fluorescence of AlexaFluor 594 (excitation: 532 nm, emission: 610 nm). **b** Growth of *E. coli* wild-type (WT) strain compared to the growth of the *ΔqueD* strain supplemented with indicated concentrations of preQ_1_-L1. **c** Relative quantification of Q-L1 (*m/z* 395 → 163, blue) in total tRNA isolated from *E. coli* WT, *ΔqueD*, *Δtgt* and *ΔqueF* cells supplemented with 5 µM preQ_1_-L1 via LC‑MS/MS. Peak areas were normalized to the UV signal of adenosine and related the signal of Q-L1 in *ΔqueD*+ 5 µM preQ_1_-L1.

**
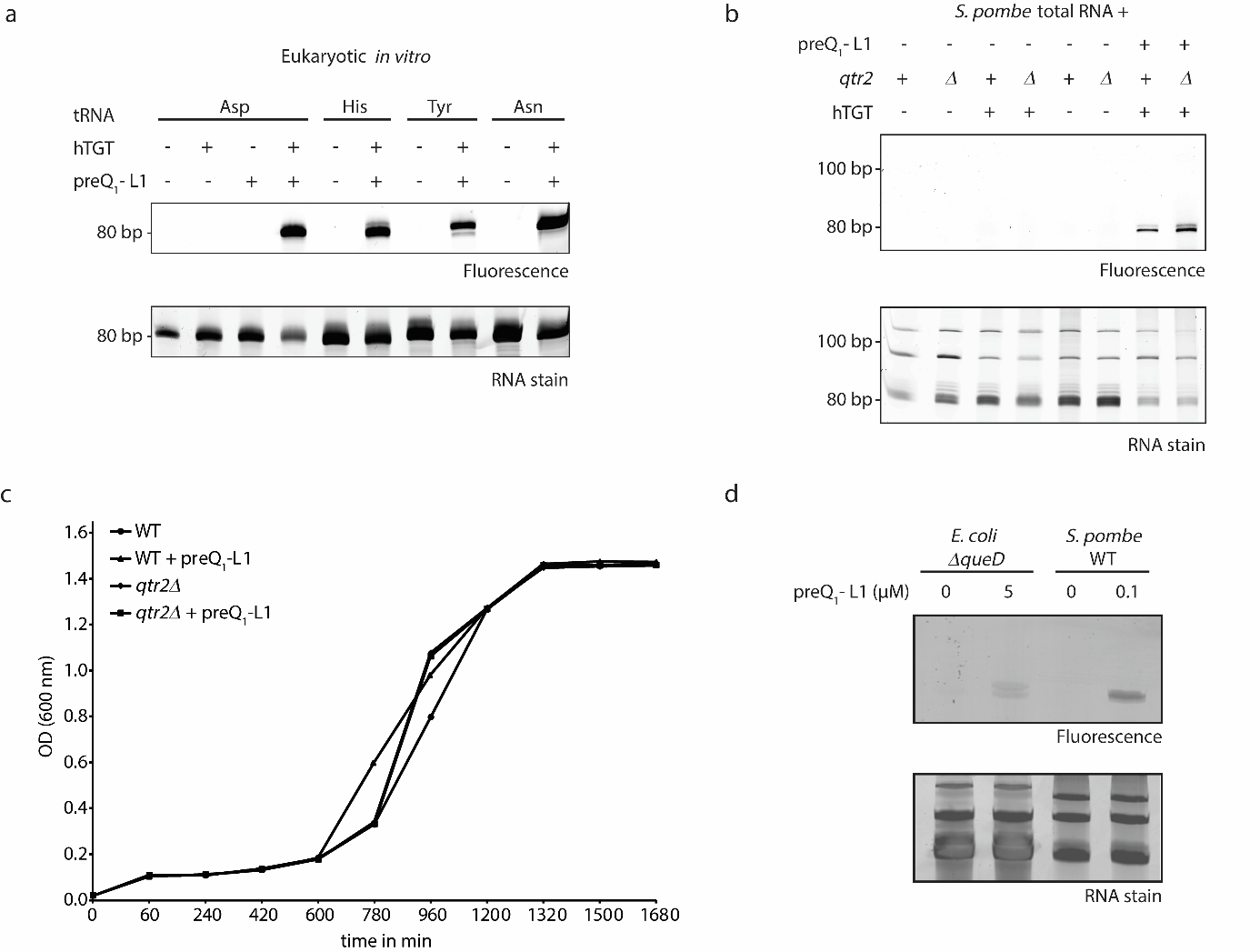
**

**Supplementary Figure 4: Incorporation of preQ_1_-L1 *in vitro* and *in vivo* in *S. pombe*. a** Human tRNA guanine transglycosylase (hTGT) was incubated with respectively indicated *in vitro* transcribed *S. pombe* tRNA in presence of preQ_1_-L1. After click reaction with AlexaFluor 594, the tRNAs were separated on a 10% polyacrylamide/ 8 M urea gel and visualized by scanning for fluorescence at 532 nm. Untreated tRNAs and tRNA^Asp^ incubated with hTGT or preQ_1_-ligand L1, respectively, served as controls. A loading control was obtained by RNA staining with SybrGold. **b** Analysis of the total RNA click product after human tRNA guanine transglycosylase (hTGT)-catalysed incorporation of preQ_1_-ligands L1 into RNA from *S. pombe* by denaturing PAGE and visualization by fluorescence scan for AlexaFluor 594 (excitation: 532 nm, emission: 610 nm). Total RNA was extracted from *S. pombe* WT cells containing functional TGT (+) and *qtr2Δ* cells that lack functional TGT (*Δ*), which were both cultured in the presence of queuine*.* The incubation of total RNA from WT and *qtr2Δ* cells without preQ_1_-ligand or without hTGT, respectively, served as negative controls. A loading control was obtained by RNA staining with SybrGold. **c** Growth of *S. pombe* wild-type (WT) and *qtr2Δ* strains in the presence or absence of 0.1 µM preQ_1_-L1. **d** Analysis of total tRNA from *E. coli* *ΔqueD* and *S. pombe* WT cells grown with the indicated concentrations of preQ_1_-L1 after click reaction by denaturing PAGE and subsequent scanning for fluorescence of AlexaFluor 594. A loading control was obtained by RNA staining with GelRed.


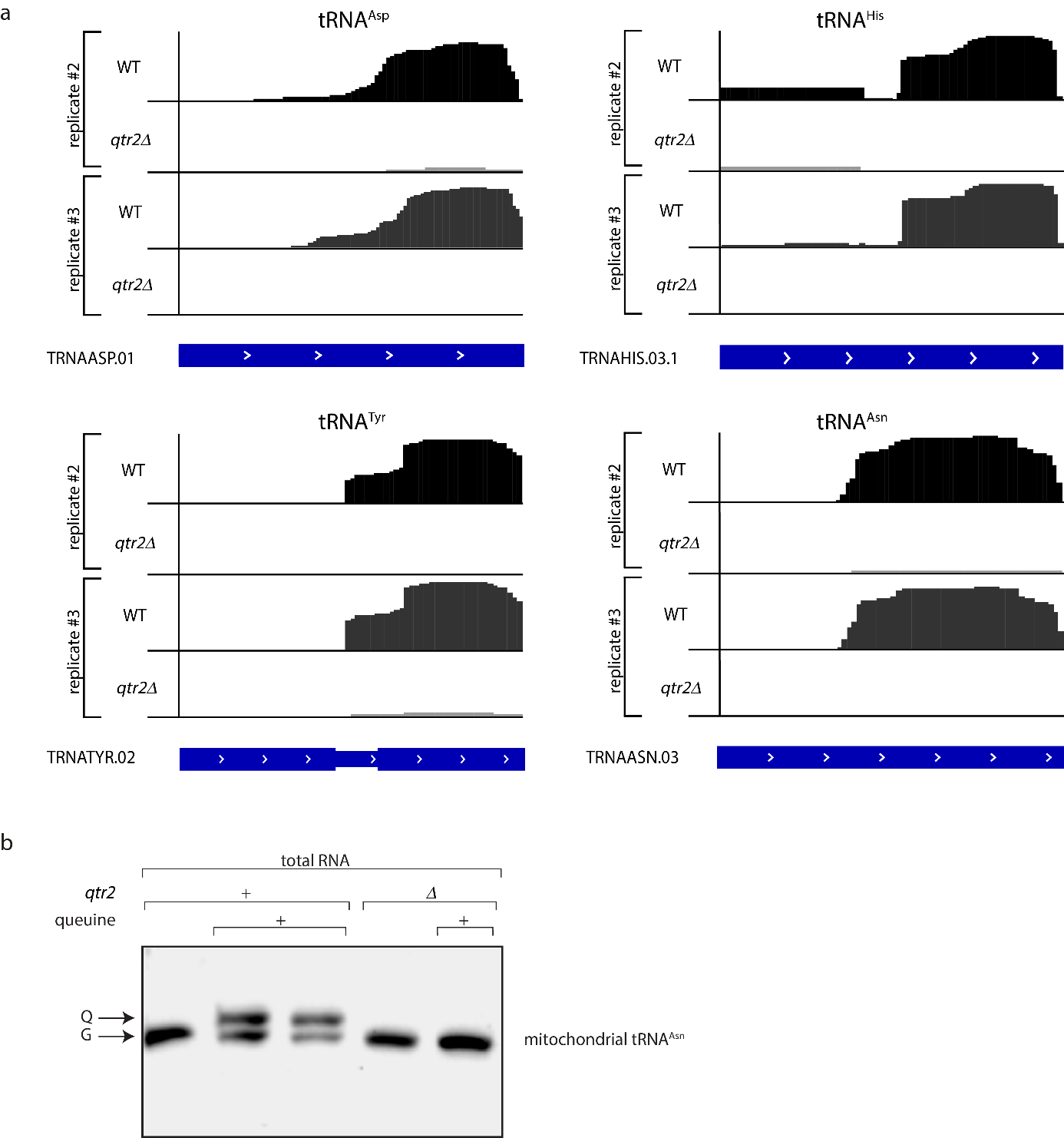


**Supplementary Figure 5: *In vivo* detection of Q-modified tRNAs in *S. pombe* using metabolic labelling with preQ_1_-L1 combined with high-throughput sequencing (Q-RIP-Seq). a** Q-RIP-Seq of tRNA^Asp^, tRNA^His^, tRNA^Tyr^ and tRNA^Asn^ after metabolic labelling with preQ_1_-L1 in *S. pombe* WT and *qtr2Δ* mutant cells and affinity purification. Coverage of the tRNA sequences from modified (WT, black) and unmodified (*qtr2Δ,* grey) samples is shown. The transcript architecture is shown below with thin and thick parts representing introns and mature tRNA sequences. Replicates #2 and #3 (c.f. Figure 5 for replicate #1) of three independent experiments are shown. Plots were generated using IGV. **b** Measurement of Q levels in mitochondrial tRNA^Asn^ using polyacrylamide gels covalently linked with N-acryloyl-3-aminophenylboronic acid (APB). Northern blotting with APB-gels was performed, and membranes were probed for mitochondrial tRNA^Asn^. RNA samples from WT and *qtr2Δ* strains cultured with or without queuine are shown. The arrows indicate the migration distance of unmodified (G) and Q-modified (Q) tRNA, respectively.


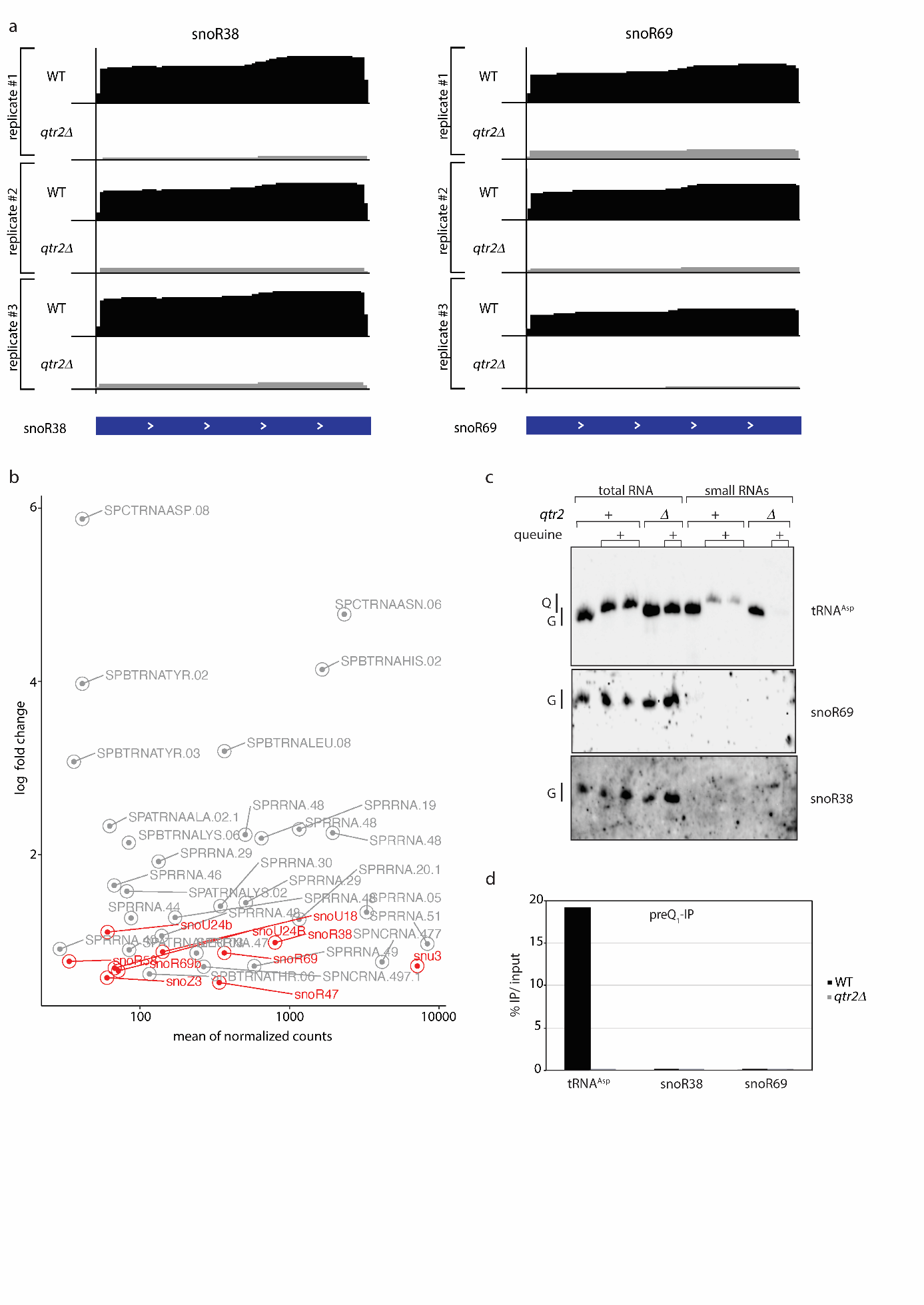


**Supplementary Figure 6: Enrichment of snoRNA sequences of *S. pombe* in Q-RIP-Seq.** RNAs that were metabolically labelled with azido-propyl-preQ_1_ were bioconjugated with biotin-alkyne, enriched with streptavidin-coated magnetic beads and subjected to HTS as in Figure 5. **a** Coverage of the snoR38 and snoR69 sequences from WT ant *qtr2Δ* *S. pombe* RNAs are shown (three independent replicates). **b** Log2 fold change of normalized read counts of RNAs from WT compared to *qtr2Δ* determined by exomePeak2. Red: snoRNAs. **c** Measurement of Q levels in tRNA^Asp^, snoR69 and snoR38 using polyacrylamide gels covalently linked with N-acryloyl-3-aminophenylboronic acid (APB). Northern blotting with APB-gels was performed, and membranes were probed with the indicated probes. RNA samples from WT and *qtr2Δ* strains cultured with or without queuine are shown. The label indicates the migration distance of unmodified (G) and Q-modified (Q) tRNA, respectively. **d** Measurement of percentage of IP to input (%IP) for tRNA^Asp^, snoR69 and snoR38 using qRT-PCR. RNAs from WT (black) and *qtr2Δ* (grey) were subjected to the Q-RIP method (IP) or only biotin-clicked (input).


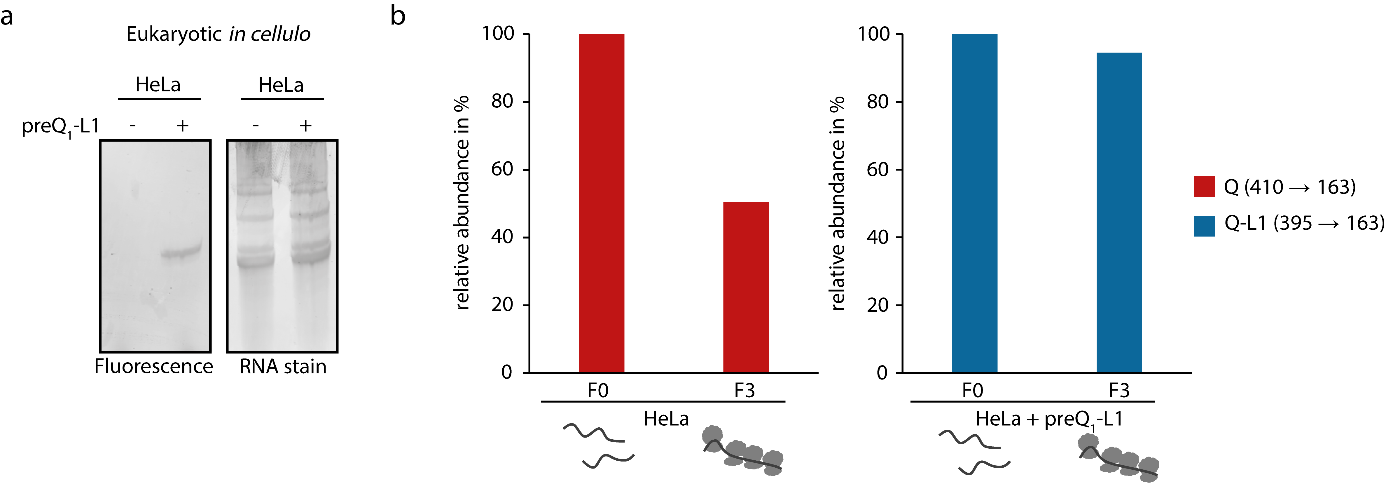
**Supplementary Figure 7: *In cellulo* incorporation of preQ_1_-L1 in HeLa cells and analysis of tRNA purified from HeLa cell polysome preparations.** A single experiment is shown. **a** Analysis of total RNA from HeLa cells grown either in q-containing DMEM medium (-) or in q-free medium supplemented with 0.1 µM preQ_1_-L1 (+) after click reaction by denaturing PAGE and subsequent scanning for fluorescence of AlexaFluor 594 (excitation: 532 nm, emission: 610 nm). b Relative quantification of Q (*m*/*z* 410 → 163, red) and Q-L1 (*m*/*z* 395 → 163, blue) in tRNA purified from fractions F0 and F3 of HeLa cells grown either in q-containing DMEM medium (HeLa) or in q-free medium supplemented with 0.1 µM preQ_1_-L1 (HeLa + preQ_1_-L1) via LC‑MS/MS. Peak areas were normalized to the UV signal of adenosine and related to the respective F0 fraction.

**Supplementary References**

1. Müller, M., Hartmann, M., Schuster, I., Bender, S., Thüring, K.L., Helm, M., Katze, J.R., Nellen, W., Lyko, F. and Ehrenhofer-Murray, A.E. (2015) Dynamic modulation of Dnmt2-dependent tRNA methylation by the micronutrient queuine, *Nucleic acids research,* **43,** 10952–10962. First published on Sep 30, 2015.
2. Becker, M., Müller, S., Nellen, W., Jurkowski, T.P., Jeltsch, A. and Ehrenhofer-Murray, A.E. (2012) Pmt1, a Dnmt2 homolog in Schizosaccharomyces pombe, mediates tRNA methylation in response to nutrient signaling, *Nucleic acids research,* **40,** 11648–11658. First published on Oct 15, 2012.
3. Gerber, H.-D. and Klebe, G. (2012) Concise and efficient syntheses of preQ1 base, Q base, and (ent)-Q base, *Organic & biomolecular chemistry,* **10,** 8660–8668.
4. Johannsson, S., Neumann, P. and Ficner, R. (2018) Crystal Structure of the Human tRNA Guanine Transglycosylase Catalytic Subunit QTRT1, *Biomolecules,* **8.** First published on Aug 24, 2018.
